# Assessing mental imagery in clinical psychology: A review of imagery measures and a guiding framework

**DOI:** 10.1016/j.cpr.2012.09.001

**Published:** 2013-02

**Authors:** David G. Pearson, Catherine Deeprose, Sophie M.A. Wallace-Hadrill, Stephanie Burnett Heyes, Emily A. Holmes

**Affiliations:** a School of Psychology, University of Aberdeen, UK; b School of Psychology, University of Plymouth, UK; c MRC Cognition and Brain Sciences, Cambridge, UK; d Department of Experimental Psychology, University of Oxford, UK

**Keywords:** Mental imagery, Working memory, Psychopathology, Autobiographical memory, Psychological assessment

## Abstract

Mental imagery is an under-explored field in clinical psychology research but presents a topic of potential interest and relevance across many clinical disorders, including social phobia, schizophrenia, depression, and post-traumatic stress disorder. There is currently a lack of a guiding framework from which clinicians may select the domains or associated measures most likely to be of appropriate use in mental imagery research. We adopt an interdisciplinary approach and present a review of studies across experimental psychology and clinical psychology in order to highlight the key domains and measures most likely to be of relevance. This includes a consideration of methods for experimentally assessing the generation, maintenance, inspection and transformation of mental images; as well as subjective measures of characteristics such as image vividness and clarity. We present a guiding framework in which we propose that cognitive, subjective and clinical aspects of imagery should be explored in future research. The guiding framework aims to assist researchers in the selection of measures for assessing those aspects of mental imagery that are of most relevance to clinical psychology. We propose that a greater understanding of the role of mental imagery in clinical disorders will help drive forward advances in both theory and treatment.

## Highlights

► Mental imagery is of potential interest and relevance across clinical disorders. ► We highlight the key domains and measures for assessing mental imagery. ► We propose a guiding framework for the selection of measures in clinical research. ► Exploring mental imagery will help drive forward advances in theory and treatment.

## 1 Introduction

Mental imagery is the simulation or re-creation of perceptual experience (Kosslyn, Ganis, & Thompson, 2001; Pearson, 2007) across sensory modalities. Such imagery has been shown to play a key role in various psychological disorders, including post-traumatic stress disorder (PTSD) (Holmes, Grey, & Young, 2005), social phobia (Hackmann, Clark, & McManus, 2000; Hirsch, Clark, & Mathews, 2006), prospective imagery in schizophrenia (D'Argembeau, Raffard, & Van der Linden, 2008), and depression (Patel et al., 2007). We have argued that the exploration of mental imagery represents a new and important arena within clinical psychopathology (Hackmann, Bennett-Levy, & Holmes, 2011; Hackmann & Holmes, 2004; Holmes & Mathews, 2010; Pearson, 2007, 2012).

The main purpose of the present review is to provide a broad framework for clinical researchers from which they can select useful measures to assess the key domains of mental imagery in clinical psychology. The experimental psychology literature on mental imagery is extensive and a considerable array of different measures have been reported to measure different abilities within the sphere of mental imagery (Logie, 1995; Pearson, 2007; Pearson, De Beni, & Cornoldi, 2001). However, surprisingly few reviews have been conducted of these measures to date, and those which have been published tend to be limited to only one or two aspects of imagery ability. For example, the review by White, Sheehan, and Ashton (1977) surveys only self-report measures of imagery, while that by McAvinue and Robertson (2008) focuses only on measures of motor imagery. A further review by McAvinue and Robertson (2007) explores self-report and objective measures but focuses on visual imagery and does not consider the clinical relevance of the measures. To our knowledge, no English-language review has yet been conducted of the key domains of mental imagery and the associated experimental tasks which may be most relevant in the field of clinical psychology. There is a need to establish the primary cognitive and clinical domains of mental imagery of interest to clinicians and researchers. There is also a need to identify the tasks which may be used to establish the extent to which any of these mental imagery domains may be impaired, distorted or even enhanced in different psychological disorders.

Mental imagery has featured prominently in current theoretical accounts of disorders such as PTSD (Brewin, Dalgleish, & Joseph, 1996; Ehlers & Clark, 2000), social phobia (Clark & Wells, 1995; Rapee & Heimberg, 1997), and bipolar disorder (Holmes, Geddes, Colom, & Goodwin, 2008). Mental imagery processes may also underlie the effectiveness of clinical treatments such as “imagery re-scripting” in Cognitive Behaviour Therapy (e.g. Holmes, Arntz, & Smucker, 2007), schema focussed therapy (e.g. Giesen-Bloo et al., 2006), and cognitive bias modification training (e.g. Holmes, Mathews, Dalgleish, & Mackintosh, 2006). The rationale for conducting the current review is therefore that a more thorough assessment of mental imagery in clinical psychology will help advance understanding of underlying mental imagery processes across a range of psychological disorders, and this in turn will help drive forward advances in both theory and treatment. In this review we discuss the key processes described in the computational theory of imagery proposed by Stephen Kosslyn and colleagues, and consider the experimental tasks that may be used to effectively assess these processes (Kosslyn, 1980, 1994; Kosslyn, Thompson, & Ganis, 2006). The computational theory was derived from mainstream experimental psychology to account for the broader process of mental imagery; therefore, it does not specifically address emotional aspects of mental imagery, but rather the everyday use and experience of mental imagery. These processes consist of image *generation*; image *maintenance*; image *inspection*; and image *transformation*. In Section 2 we outline our strategy for conducting the literature review and describe selection criteria adopted for inclusion of different mental imagery measures. In Section 3 we discuss the experimental literature in relation to four main stages of mental imagery (generation, maintenance, inspection, and transformation), and in relation to its general use and experience. In Section 4 we review recent research in clinical psychology that has utilised mental imagery measures in the assessment of different psychological disorders. In Section 5 we critically review measures and procedures judged most relevant for assessment of key domains of mental imagery, and from which clinicians and experimental psychologists may select tasks as appropriate for the population under investigation. Finally, in Section 6 we propose a guiding framework that highlights the broad domains of imagery assessment shown to be most important in research to date.

## 2 Method

### 2.1 Literature search strategy

Published studies were identified through searches of Psychological Abstracts (PsycINFO) and ISI Web of Knowledge (All databases) using keyword, title and abstract information. The initial search term ‘mental imagery’ returned 8838 articles. The search was then further refined using the terms ‘imagery measures’; ‘imagery assessment’; ‘imagery’ combined with ‘clinical’; and ‘imagery’ combined with ‘subjective’. ‘Imagery’ and ‘image’ were also searched in combination with ‘generation’, ‘maintenance’, ‘inspection’, and ‘transformation’. Related searches were carried out using the terms ‘visual working memory’ and ‘spatial working memory’ based on evidence for overlap between these constructs and mental imagery processes (for discussion see Section 3.2). In addition, the lists of references from review papers, book chapters, and other relevant articles were consulted to identify further items. Only English-language articles were considered. Unpublished studies or studies published in non peer-reviewed journals were excluded from the search.

All relevant peer-reviewed published studies in English were then evaluated for inclusion. The criteria adopted for selecting imagery measures were based on (a), whether a study assessed a specific cognitive stage of mental imagery (as defined in Section 3); and/or (b), whether a study reported significant findings for mental imagery within the context of clinical psychology (as outlined in Section 4). Evaluation using these criteria identified 65 relevant studies presenting measures assessing specific cognitive stages of imagery (Table 1), 19 studies with measures assessing general imagery use and experience (Table 2), and 28 studies with measures assessing specific clinical aspects of mental imagery (Table 3).

### 2.2 Inclusions and omissions

In an attempt to balance critical thoroughness with overall manageability our review focuses predominantly on mental imagery assessment in relation to PTSD, schizophrenia, social phobia, depression, and bipolar disorder (as discussed in Section 4). These disorders are not presented as exhaustive, and are highlighted on the basis that the literature associated with the disorders has particular relevance for evaluating the importance of mental imagery assessment in clinical psychology. We acknowledge there are other clinical disorders which could be discussed in relation to mental imagery, including craving (May, Andrade, Panabokke, & Kavanagh, 2004), stroke (Nilsen, Gillen, DiRusso, & Gordon, 2012), multiple sclerosis (Heremans et al., 2012) and eating disorders (Tatham, 2011), amongst others.

Our review also focuses on providing an integrative analysis of studies dealing with the visual and spatial aspects of mental imagery, as historically these are the domains most extensively researched across the imagery literature (Kosslyn et al., 2006; Pearson, 2007; Pearson et al., 2001). Practical considerations mean that assessment of the literature on mental motor imagery is beyond the scope of the current review, although we recognise its importance in relation to clinical disorders such as anorexia nervosa (Guardia et al., 2010), stroke (Liepert, Greiner, Nedelko, & Dettmers, 2012), and spastic hemiplegia (Williams, Anderson, Reid, & Reddihough, 2012). We note that existing reviews of the mental motor imagery literature have been provided by McAvinue and Robertson (2008), Holmes and Calmels (2008) and Olsson and Nyberg (2010).

## 3 Cognitive stages of mental imagery

This section describes cognitive stages of mental imagery derived from a computational theory of imagery and high-level visual perception proposed by Stephen Kosslyn and colleagues (Kosslyn, 1980, 1994; Kosslyn et al., 2006). Following the computational approach described by Marr (1982), Kosslyn's theory aims to establish which cognitive systems and processes are necessary in order to produce the patterns of behaviour associated with the generation and manipulation of mental images. The computational approach aims to understand cognition in terms of the processing subsystems that carry out data-transforming computations in a “systematic, informationally interpretable way” (Kosslyn, 1987, p. 150). The model provides a comprehensive account of the processes and subsystems that underlie the functioning of mental imagery in the brain, and has been widely influential in experimental cognitive psychology, and in neuropsychological investigations of how imagery maps on to different structures of the brain (Cichy, Heinzle, & Haynes, 2012; Slotnick, Thompson, & Kosslyn, 2012).

### 3.1 Image generation

There are two distinct routes by which mental imagery can be created within consciousness (Pearson, 2007). First of all an image can be created directly from immediate perceptual information. For example, someone can look at a picture of a horse, create a mental image of the picture in their mind, and then maintain this mental image as they look away or close their eyes. Second, an image can be created entirely from previously stored information held in long-term memory. For example, someone can hear the word “horse” and then create mental imagery based on their previous experience of what a horse looks like. Previous studies have demonstrated that there can be significant cognitive differences in performance between imagery generated from either short-term or long-term memory (Hitch, Brandimonte, & Walker, 1995; Pearson & Logie, 2004). For example, Cornoldi, De Beni, Guisberti, and Massironi (1998) report that the colour of geometric patterns is less accurately retained in mental images generated from long-term memory in comparison to short-term memory. While visual and auditory mental images are usually reported as being the most frequently experienced (Betts, 1909; Tiggemann & Kemps, 2005), other sensory modalities include olfactory imagery (Stevenson & Case, 2005), gustatory imagery (Tiggemann & Kemps, 2005), and haptic imagery (Juttner & Rentschler, 2002).

Imagery can result from both deliberate and involuntary recall processes; in experimental psychology the focus has typically been on deliberately generated imagery whereas in psychopathology, the focus has typically been on involuntary imagery which comes to mind unbidden. According to the computational theory of imagery proposed by Kosslyn (1980, 1994); Kosslyn et al., 2006) voluntary mental imagery is often generated in order to make explicit geometric properties of an object such as its size. For example, if asked to decide whether an elephant possesses a long or short tail many people report deliberately visualizing the appearance of an elephant from memory (Farah, Hammond, Levine, & Calvanio, 1988). Mental images are generated within a topographically organised area of the brain known as the *visual buffer* (Kosslyn, 1980, 1994; Kosslyn et al., 2006). Different categories of imagery can also be generated, such as general images, specific images, and autobiographical or episodic images (Cornoldi & Rossana, 1998; Gardini, Cornoldi, De Beni, & Venneri, 2009). Studies also suggest that the generation of images of whole objects may engage different processes than imagining specific parts of an object (Farah, 1988; Li et al., 2010). Deficits in image generation ability have been linked to conditions including representational neglect (Palermo, Piccardi, Nori, Giusberti, & Guariglia, 2010), congenital blindness (Eardley & Pring, 2006), depression (Zarrinpar, Deldin, & Kosslyn, 2006), and attention deficit hyperactivity disorder (Abraham, Windmann, Siefen, Daum, & Gunturkun, 2006).

### 3.2 Image maintenance

Once generated, a mental image is subject to rapid decay with an average duration of only 250 ms, which corresponds to the time necessary to make an eye movement (Kosslyn, 1994). This brief duration means that active maintenance of the image is required in order for any inspection or transformation processes to be performed. In Kosslyn's computational theory of imagery, such maintenance is achieved by the re-activation of visual memory representations in an Object Properties-Processing subsystem (Kosslyn, 1980; Kosslyn et al., 2006). This subsystem is responsible for analysing object properties such as shape and colour and it plays a role during both visual perception and mental imagery. Maintenance processes have been demonstrated for mental images generated from both long-term memory (Cocude & Denis, 1988) and short-term memory (Watkins, Peynircioglu, & Brems, 1984).

Even with active maintenance people can struggle to hold a mental image clearly in mind for more than a few seconds (Cocude, Charlot, & Denis, 1997; Cocude & Denis, 1988; Pazzaglia & Cornoldi, 1999). Kosslyn et al. (2006) have argued that this brief duration of generated images results from them sharing the same topographically organised areas of occipital cortex that are used during visual perception. One consequence of this shared neural substrate is the occurrence of mutual interference between visual imagery and visual perception, with several studies demonstrating that imagery can be disrupted by concurrent visual processing (e.g. Baddeley & Andrade, 2000; McConnell & Quinn, 2004; Quinn & McConnell, 2006). However, it is still the case that imagery can effectively be experienced alongside competing perceptual input. For example, there is no evidence for any consistent difference in the reported vividness of images experienced under ‘eyes-open’ and ‘eyes-closed’ conditions (Isaac & Marks, 1994; McKelvie, 1995).

Image maintenance may also be dependent upon general attentional resources that become rapidly depleted (Logie, 1995; Pearson, 2007; Pearson et al., 2001). Studies have shown that the maintenance of visual mental imagery places considerable demands upon general-purpose attentional resources (Logie & Salway, 1990; Pearson, Logie, & Green, 1996; Salway & Logie, 1995). Based on this, Pearson, Logie, and Gilhooly (1999) have argued that image maintenance may better be considered a function of the central executive component of working memory rather than as a separate visually-based process. Some evidence suggests that even involuntarily experienced mental images place demands upon general attention-based working memory resources. An early study conducted by Bexton et al. showed that involuntary visual imagery associated with sensory deprivation could be dispelled by asking participants to perform demanding cognitive tasks such as mental arithmetic (Bexton, Heron, & Scott, 1954). More recently concurrent mental arithmetic has also been shown to significantly reduce the experienced vividness and emotionality of mental images for a traumatic memory (Bexton et al., 1954; Engelhard, van den Hout, & Smeets, 2011). Indeed, in clinical practice, involuntary mental images are considered ‘intrusive’ by virtue of being unwanted and unbidden, such is their ability to ‘hijack attention’ (Berntsen, 2001; Clark, Holmes, & Mackay, in press).

The relationship between the maintenance of visual mental imagery and the retention of information within visual short-term memory is currently unclear, and the two processes may be related or even synonymous with each other. According to Kosslyn et al., visual mental imagery and visual short-term memory rely on a common ‘depictive representation’ system, such that visual mental imagery is disrupted by maintenance of perceptually similar items within visual short term memory (Borst, Ganis, Thompson, & Kosslyn, 2011). However, some evidence suggests that the conscious experience of mental imagery and short-term visuo-spatial retention can be dissociated from each other, implying at least a partial dissociation between the cognitive processes that underlie each function (Pearson, 2007; Zeman et al., 2010). In addition, while the majority of the research literature has tended to focus on the maintenance of visual mental images, imagery in other sensory modalities also necessitates a maintenance process prior to any further inspection or transformation processes being carried out (Zatorre, Halpern, & Bouffard, 2010). This is because mental images usually fade quickly once generated in order to avoid any disruption to normal perception (Kosslyn, 1980, 1994; Kosslyn et al., 2006).

Based on evidence that visual mental imagery and visual short-term memory may rely upon a common ‘depictive representation’ system, administering tasks developed to test retention in visual short-term memory may shed light on the capabilities of this system for holding visual mental images. Similarly, since active maintenance of visual mental images requires the central executive component of visual working memory, ‘complex span’ tasks might be used to assess an individual's capacity to effortfully maintain visual mental images.

### 3.3 Image inspection

Once generated and maintained, a mental image can be *inspected* to provide a basis for further cognitive processing. The inspection process involves interpreting an object-based characteristic or spatial property of a generated image. For example, a participant might be asked to describe the shape formed by a fox's ear. Typically in response to this type of question a person would generate a mental image of a fox and then examine the shape of the ears within the image in order to produce a response (Kosslyn et al., 2001). Within the computational theory such image inspection processes involve shifting an attention window across the mental image held within the visual buffer to encode its geometric properties (Kosslyn, 1994; Kosslyn et al., 2006). The attention window typically shifts across an image in incremental stages, with the spatial relationships between different parts of an image encoded by a subsystem dedicated to processing spatial properties (Kosslyn, 1994). Studies based on examining regional cerebral blood flow suggest that the processes that contribute to image inspection are distinct from those for image generation, maintenance, and transformation (Kosslyn et al., 2004).

One of the most widely researched aspects of image inspection is mental scanning during which the focus of attention in an image is shifted from one point to another. A large number of studies suggest that the time taken to incrementally scan across a mental image increases linearly with the distance scanned (Denis & Kosslyn, 1999). Early scanning paradigms involved giving participants explicit instructions to scan across a mental image, but these findings were criticised as potentially reflecting participants' prior expectations of simulating movement rather than underlying spatial properties of the image itself (e.g., Baddeley, 1990; Denis & Carfantan, 1990; Pylyshyn, 1981). However, more recent scanning paradigms do not make any explicit reference to scanning across a mental image (e.g., Borst & Kosslyn, 2010; Dror, Kosslyn, & Waag, 1993), making such a ‘tacit knowledge’ criticism harder to apply to the experimental findings (Kosslyn et al., 2006). Inspection processes can be applied both to imagery that derives from perceptual experience and to images that are created from verbal descriptions (Pearson et al., 2001). However, a PET study conducted by Mellet et al. (2002) suggests there may be significant differences in scanning of images that derive either from verbal descriptions or directly from visual learning. Mental scanning has been demonstrated in congenitally blind participants, although reported scanning times are shorter in duration in comparison to sighted groups (Iachini & Ruggiero, 2010).

### 3.4 Image transformation and manipulation

The active manipulation and transformation of mental images occurs during many different aspects of everyday problem-solving and creative thinking (Pearson, 2007; Pearson et al., 2001). One of the most extensively researched types of image transformation is that of *mental rotation*, a paradigm first established by Shepard and Metzler (1971). The standard finding is that the further a participant has to mentally rotate an image the longer it takes them to make a response (Shepard & Cooper, 1982). Within the computational model of imagery mental rotation occurs through an incremental modulation of the mapping function between the object-properties-processing subsystem and the visual buffer in which the image is represented (Kosslyn et al., 2006).

Another form of transformation is *image restructuring* in which the interpretation of a mental image is changed or modified in some way (Finke, Pinker, & Farah, 1989; Verstijnen, Hennessy, van Leeuwen, Hamel, & Goldschmidt, 1998). This type of transformation underlies performance during component detection tasks (Reed & Johnson, 1975) or during the re-interpretation of ambiguous figures using imagery alone (Mast & Kosslyn, 2002; Riquelme, 2002). These types of transformations can be constrained by the contextual reference frame within which an image is originally generated (Reisberg, 1996; Reisberg & Logie, 1993; Verstijnen, Hennessy et al., 1998; Verstijnen, van Leeuwen, Goldschmidt, Hamel, & Hennessey, 1998), and by verbal overshadowing effects which reflect the interference of visual memory as a result of verbally describing the same stimuli (Brandimonte & Collina, 2008). Additional image transformations can include changes in size (Kosslyn, 1975) and colour (Watkins & Schiano, 1982).

A combination of mental rotation and image restructuring occurs during *mental synthesis*, in which discrete parts of an image are transformed and manipulated in order to produce novel patterns or allow new insights (Pearson et al., 2001). Such synthesis has been linked to cognitive activities such as design (Reed, 1993), scientific reasoning (Gardner, 1953), and general problem-solving (Antonietti & Baldo, 1994). Though less formally studied, this process clearly relates to the clinical psychology technique of ‘imagery restructuring’ or ‘imagery re-scripting’ as used in Cognitive Behaviour Therapy (Arntz, 2012; Hagenaars & Arntz, 2012; Holmes, Arntz et al., 2007).

Some research findings suggest that mental transformation of a perceived object (such as mental rotation) can be dissociated from transformations of larger environmental scenes (Hegarty & Waller, 2004; Kozhevnikov & Hegarty, 2001) and from visualizing spatial locations (Thompson, Slotnick, Burrage, & Kosslyn, 2009). Research also shows that mental transformations can become impaired in conditions such as hemispatial neglect (Palermo et al., 2010) and developmental coordination disorder (Williams, Thomas, Maruff, & Wilson, 2008). While the majority of research findings on image transformation relate to visual mental imagery, there is evidence to support transformations in other sensory modalities such as auditory imagery (Zatorre et al., 2010) and haptic imagery (Miquee et al., 2008).

### 3.5 Subjective experience of mental imagery

The measures described in the previous sections focus on mental imagery as internal representations that reproduce or simulate the properties of perceptual representations (Pearson, 2007). However, these measures do not address the *phenomenological* characteristics of mental imagery, that is, how mental imagery is consciously experienced. This aspect of imagery can be assessed using subjective measures in which participants rate or describe different characteristics of their conscious experience of mental imagery. One common dimension assessed by subjective measures is the *vividness* of a mental image, which can be used to refer both to luminosity and clarity of mental imagery, as well as the extent to which an individual's subjective experience of imagery is similar to actual perceptual experience (Pearson et al., 2001). The extent of mental imagery experienced in everyday life has also been linked to individual differences in areas such as memory and creative thinking (Pearson, 2007; Valenti, Libby, & Eibach, 2011).

Baddeley and Andrade (1998) have argued that image vividness reflects the storage of rich and detailed sensory-based representations held in the visual and auditory slave systems of working memory. This hypothesis is supported by the results of an fMRI study conducted by Belardinelli et al. (2009) which found that vividness of mental imagery in different modalities is related to higher activation in neural substrates associated with perception of the same modality. Modality-specific visual and auditory neural substrates have also been linked to the subjective experience of mental imagery and to the retrieval of sensory-bound information from autobiographical memory (Huijbers, Pennartz, Rubin, & Daselaar, 2011). There is an emerging literature on vividness as probed via neuroimaging studies e.g. by Stokes and colleagues (Stokes, Thompson, Cusack, & Duncan, 2009) .

Consistent with these findings are behavioural studies that show concurrent tasks placing a high load on working memory capacity can disrupt the vividness of mental imagery for negative autobiographical events (e.g., Andrade, Kavanagh, & Baddeley, 1997; Kavanagh, Freese, Andrade, & May, 2001; van den Hout, Muris, Salemink, & Kindt, 2001). However, it remains a source of debate in the literature whether such disruption reflects modality-specific interference or general working memory load. For example, in a recent study by van den Hout et al. (2011), both EMDR (Eye Movement Desensitization and Reprocessing) and attentional breathing exercises associated with Mindfulness-Based Cognitive Therapy (MBCT) were found to reduce vividness and emotionality of negative memories. Van den Hout et al. interpret this finding in terms of task performance of EMDR and MBCT both equally taxing the limited capacity of the working memory system. In contrast, other studies have found concurrent visuo-spatial tasks are more effective at reducing subjective vividness and emotionality than comparable verbal tasks (e.g., Lilley, Andrade, Turpin, Sabin-Farrell, & Holmes, 2009) or impact differentially on later involuntary images (Holmes, James, Kilford, & Deeprose, 2010).

A number of measures have been developed aiming to tap into individual differences in the subjective experience of mental imagery as reviewed by Richardson (1994). Although the predominant focus has typically been on the assessment of trait use of visual imagery, there have also been attempts to assess imagery both in relation to specific tasks and across sensory modalities (Section 5.5, and summarised in Table 2).

## 4 Imagery in clinical psychology

Clinical studies investigating mental imagery have employed a diverse and sometimes eclectic range of measures. Although various focuses have been on involuntary imagery, or processes of imagery thought to reflect an inherent processing bias (e.g., valence of imagery content and perspective of imagery), it has typically been held that the key facets of imagery are specific to the disorder under consideration. We will now consider the assessment of imagery in clinical psychology with specific reference to PTSD, social phobia, schizophrenia and depression and finally, bipolar disorder. Although bipolar disorder has received less attention to date in this field, we have recently proposed that imagery may play a particular role in influencing the key characteristics of the disorder (Holmes, Geddes et al., 2008).

### 4.1 Post-traumatic stress disorder

PTSD may result from experiencing or viewing a traumatic event involving death, serious injury, or threat to self or others (American Psychiatric Association, 2000). Flashbacks (intrusive images) are the hallmark symptom of this disorder (Ehlers, Hackmann, & Michael, 2004; Ehlers & Steil, 1995; Speckens, Ehlers, Hackmann, Roths, & Clark, 2007) and consist of vivid and emotional memories for the trauma, accompanied by a strong sense of current threat or “here and now-ness” (Holmes & Mathews, 2010). Current cognitive information processing theories of PTSD (Brewin et al., 1996; Ehlers & Clark, 2000) converge on the idea that intrusive images develop due to impaired information processing during the traumatic event (Holmes & Bourne, 2008). As such, many studies have explored the intrusive nature of this mental imagery in PTSD.

#### 4.1.1 Imagery assessment in PTSD

Imagery in PTSD has largely been assessed using techniques such as script-driven imagery procedures (Section 5.6.1), and intrusion diaries and interviews (Sections 5.6.4 and 5.6.5), in which the objective is to explore the content and frequency of image-based intrusions. There has been comparatively little research focusing on investigating the disorder from the perspective of cognitive behavioural imagery measures. We propose it will be valuable to establish whether PTSD groups display a similar deficit in image generation processes to that previously reported for social phobia (Morrison, Amir, & Taylor, 2011, Section 4.2.1) and depression (Cocude et al., 1997, Section 4.4.1; Zarrinpar et al., 2006). If present, such a deficit might reflect a bias towards generating trauma-related imagery that creates interference with image generation for neutral stimuli.

Some researchers have attributed the success of clinical treatments of PTSD such as EMDR to modality-specific disruption of visual working memory processes (e.g. Holmes et al., 2010; Lilley et al., 2009; van den Hout et al., 2012). This can be related to the literature on image maintenance and inspection processes described in Sections 3.2 and 3.3, which have been argued to overlap with the processes involved during rehearsal in visuo-spatial working memory (Borst et al., 2011; Logie, 1995; Pearson & Sawyer, 2011). In addition, imagery re-scripting techniques used to treat PTSD in CBT (e.g. Holmes, Arntz et al., 2007) can be understood in relation to image transformation and restructuring processes (see Sections 3.4 and 5.4.4).

### 4.2 Social phobia

In social phobia, individuals fear situations such as public speaking, interviews and meeting people they do not already know (American Psychiatric Association, 2000). The recurrent and distressing imagery reported by individuals with social phobia is often that of a previously experienced event (Hackmann et al., 2000) and such imagery is proposed to contribute to the maintenance of the disorder in contemporary cognitive models (Clark & Wells, 1995; Rapee & Heimberg, 1997). The observer perspective adopted within imagery is a feature of this disorder (Hackmann et al., 2000). Such imagery has been demonstrated to have a causal impact on anxiety (Hirsch, Clark, Williams, Morrison, & Mathews, 2005).

#### 4.2.1 Imagery assessment in social phobia

Morrison et al. (2011) have explored performance of image generation in socially anxious and non-socially anxious participants (see Section 5.1.1 for a description of the imagery procedure). They found that socially anxious participants were impaired in their ability to generate mental imagery for neutral stimuli (letters of the alphabet) as compared to non-socially anxious participants. It has yet to be established whether a similar generation deficit can be found with imagery for emotional material, or whether socially-anxious individuals may even display enhanced generation times for highly self-relevant imagery such as self-image. This highlights the need to explore underlying cognitive imagery processes in addition to the phenomenology of the imagery associated with social phobia.

Hackmann et al. (2000) have used a semi-structured interview (Section 5.5.8) to explore the frequency and nature of mental images in social phobia. All participants reported recurrent negative images, most of which were related to an adverse social event occurring near to the time of onset of the disorder suggesting a relationship with onset and maintenance of the disorder. The same interview method has been employed by Lockett et al. (2012) in relation to socially-anxious individuals with a comorbid diagnosis of psychosis. They found that while some participants reported typical social anxiety imagery others experienced more threatening imagery that may be related to residual psychotic paranoia. Furthermore, while social anxiety images were experienced typically from an observer perspective, imagery related to psychosis tended to be experienced more from a field perspective. We propose it will be valuable to explore in future research whether these differences in the phenomenology of imagery associated with comorbid conditions of social phobia and psychosis are associated with significant differences in cognitive processes such as image inspection (Section 3.3) or image manipulation (Section 3.4).

### 4.3 Schizophrenia

Schizophrenia is characterised by an array of symptoms: positive symptoms include hallucinations, delusional beliefs, disorganised speech and behaviour whereas negative symptoms include blunted emotion and lack of motivation (American Psychiatric Association, 2000). Mental imagery research in this field has focused on the positive symptom of hallucinations and the relationship with trait imagery, given the observation that hallucinatory imagery appears in the most severe form to “overtake reality” in psychosis (e.g. Holmes & Mathews, 2010; Oertel et al., 2009). The second key theme in imagery research has been the exploration of imagery of the past and for the future given the proposed disturbance of the sense of self across time in the disorder (Danion et al., 2005; D'Argembeau et al., 2008).

#### 4.3.1 Imagery assessment in schizophrenia

The Questionnaire on Mental Imagery (Sheehan, 1967; Section 5.5.1) has been used by Oertel et al. (2009) to investigate the relationship between the vividness of imagery and predisposition towards hallucinatory experience in schizophrenia. Findings revealed greater vividness of imagery in patients with schizophrenia and also in first-degree relatives and high-schizotypy controls when compared to low-schizotypy controls. On this basis, the authors suggested that vividness of imagery may be an independent symptom and trait marker for the schizophrenia spectrum. However, Bell and Halligan (2010) report a study in which the Vividness of Visual Imagery Questionnaire (Section 5.5.2.) was used to explore the relationship between vividness of imagery and schizotypal traits in an analogue non-clinical population. Interestingly, this study did not replicate the findings of Oertel et al. (2009), in that no significant differences were found between high and low schizotypy groups on imagery vividness as assessed using the VVIQ. Although explanation for the conflicting findings may be attributed to differences in the samples in each study, it is noteworthy that, as suggested by Bell and Halligan, the VVIQ specifically measures visual imagery whereas the QMI looks at imagery across seven sensory domains. Thus, demonstrations suggesting that vividness of imagery is a trait marker for schizotypy may only be observed when imagery is assessed across several domains.

Considering the reported differences in phenomenology of imagery associated with high and low schizotypy we argue this clinical group will be valuable to examine in terms of cognitive imagery processes such as image maintenance (Section 3.2) and image inspection (Section 3.3). Aleman, de Haan, and Kahn (2005) have reported that people with schizophrenia are impaired on a measure of image inspection (see Section 5.3.9), but not image generation (Section 5.1.1). This may reflect deficiency in the voluntary control of imagery, or over-taxing of imagery processes caused by persistent hallucinatory or delusional states. There is also evidence that schizophrenia is related to problems in maintaining and controlling visual information in working memory (Kang, Sponheim, Chafee, & MacDonald, 2011), as well as deficits in spatial working memory maintenance (Lee, Folley, Gore, & Park, 2008).

Assessment of mental imagery in schizophrenia must also take into account the presence of generalised cognitive deficits, in particular slowness of information processing and executive dysfunction (Rajji, Ismail, & Mulsant, 2009). Such general deficits could produce impairment across multiple imagery measures, in particular when considering that executive resources may play a central role in imagery experience (Logie & Salway, 1990; Pearson et al., 1996; Pearson et al., 1999; Salway & Logie, 1995). However, the studies reported by Aleman et al. (2005), Kang et al. (2011), and Lee et al. (2008) described above indicate that investigation of specific imagery-based deficits in schizophrenia can be valuable and informative.

### 4.4 Depression

Depression is associated with a range of cognitive (e.g., concentration difficulties), emotional (e.g., feelings of extreme sadness and hopeless), and behavioural (e.g., sleep disruption) symptoms (American Psychiatric Association, 2000). Traditionally, depression has been associated with verbal, rather than imagery based processes, such as negative rumination (Fresco, Frankel, Mennin, Turk, & Heimberg, 2002). However, a role for imagery has more recently become of concern, with up to 90% of depressed patients reporting distressing intrusive memories of past experiences (Birrer, Michael, & Munsch, 2007), see also Reynolds and Brewin (1998). Furthermore, negative, maladaptive appraisals of intrusive memories, (e.g., ‘having this memory means that I am weak’) have been proposed to maintain the occurrence of intrusive memories, and in turn, depressive symptoms (Starr & Moulds, 2006; Williams & Moulds, 2008). Thus studies in depression have typically investigated negative imagery of the past. More recently there has been interest in a lack of positive imagery for the future (Holmes, Lang, Moulds, & Steele, 2008; Morina, Deeprose, Pusowski, Schmid, & Holmes, 2011; Werner-Seidler & Moulds, 2010) and image-based interpretation biases (Holmes, Lang, & Deeprose, 2009).

#### 4.4.1 Imagery assessment in depression

The Image Duration Task (seeSection 5.2.6) has been used to compare the latency and duration of images in depressed participants compared to healthy volunteers (Cocude et al., 1997). Although the depressed participants found it more difficult to generate images (e.g. 2.61 seconds for mean high-imagery noun generation time in the control group, compared to 4.43 seconds in the participants with depression), the two groups did not differ in the duration of images generated. This suggests impairment in image generation processes rather than image maintenance. The mental rotation of letters (see Section 5.4.1.1.) has also been shown to be impaired in unipolar major depression (Rogers et al., 2002), which may be indicative of a more general deficit in image transformation processes (Section 3.4).

For associated phenomenology the mental imagery interview (Section 5.5.8) has been used to explore the frequency and nature of suicidal imagery in depression. Results showed that all participants experienced intrusive, recurrent suicide-related images when at their most despairing, which the authors termed “flash-forwards” to suicide (replicated by Crane, Shah, Barnhofer, & Holmes, 2012; Hales, Deeprose, Goodwin, & Holmes, 2011). However, using the Future Cueing Task (Section 5.7.2), Williams et al. (1996) found that suicidal depressed participants demonstrated impairment in generating images of the future for all sets of cues (positive, negative and neutral) compared to matched controls. This degree of impairment was found to correlate with the extent to which participants had difficulty in retrieving specific autobiographical memories.

As with schizophrenia (Section 4.3), assessment of imagery in patients suffering from depression should also take into account the presence of generalised cognitive deficits (Castaneda, Tuulio-Henricksson, Marttunen, Suvisaari, & Lönnqvist, 2008).

### 4.5 Bipolar disorder

Bipolar disorder is defined by manic episodes interspersed with episodes of depression (American Psychiatric Association, 2000). As many as 90% of bipolar patients experience a comorbid anxiety disorder at some stage (Merikangas et al., 2007) and the disorder presents a major cause of mortality due to the prevalence of suicide (Balazs et al., 2006; Hawton, Sutton, Haw, Sinclair, & Harriss, 2005). A recent cognitive model by Holmes, Geddes et al. (2008) suggests that bipolar disorder may be associated with particularly high imagery-proneness and proposes that imagery may play a role in modulating the mood swings which characterise the disorder. The model suggests that prospective imagery may be of particular interest in bipolar disorder, and may contribute towards not only depressive episodes but mania as well.

#### 4.5.1 Imagery assessment in bipolar disorder

In bipolar disorder, significantly higher Spontaneous Use of Imagery Scale (see Section 5.5.3) scores have been observed compared to healthy volunteers (Holmes et al., 2011) and unipolar depressed participants (Hales et al., 2011), indicating higher general use of imagery in bipolar patients. Patients with bipolar disorder have also shown significantly higher ratings on the imagery processing item of the Verbal and Imagery-based Thoughts Visual Analogue Scale (see Section 5.5.7) compared to healthy volunteers and in contrast, significantly lower ratings on the verbal processing item (Holmes et al., 2011). Convergent findings have been reported by McCarthy-Jones, Knowles, and Rowse (2012) using a bespoke scale of imagery use.

The Mental Imagery Interview (Section 5.5.8) has been used to investigate imagery relating to suicidal cognitions in both bipolar and unipolar patients (Hales et al., 2011). All participants reported imagery relating to suicide, thus replicating previous findings in unipolar depression (Holmes, Crane, Fennell, & Williams, 2007) and extending these to bipolar disorder. There were no differences between the bipolar and unipolar group in terms of verbal cognitions related to suicide. However, the bipolar group rated their suicidal imagery as significantly more compelling, reported a significantly greater preoccupation with the suicidal imagery and finally, were more than twice as likely to report that the images made them want to take action to complete suicide than the unipolar group.

In terms of cognitive imagery processes recent findings from Pan, Hsieh, and Liu (2011) suggest that remitted bipolar patients show deficits in visuospatial working memory under high working memory load conditions. There is thus a need to further explore bipolar disorder in relation to the experimental measures of imagery performance (see also Thompson et al., 2005). As in depression and schizophrenia, general cognitive deficits should also be taken into account when assessing mental imagery in bipolar patients (Bearden, Hoffman, & Cannon, 2001).

## 5 A critical review of mental imagery measures in clinical and experimental psychology

This section reviews experimental and clinical measures for assessing mental imagery reported in the literature. The basis for the literature search and criteria for task selection are outlined in Section 2. Table 1 summarises the measures for assessing the cognitive stages of imagery as outlined in Section 3. Table 2 summarises measures for assessing general imagery use and experience. Finally, Table 3 summarises measures that have specific focus on imagery use in relation to clinical disorders. The original references for tasks are identified along with published variants. The format of tasks (computer-based or pen-and-paper) is also noted.

### 5.1 Experimental tasks for image generation

#### 5.1.1 Image generation task

Podgorny and Shepard (1978) devised an image generation procedure in which participants are required to imagine a pattern of shaded blocks projected onto a blank 5 × 5 matrix (either following visual presentation of the pattern or following a series of verbal instructions). On each experimental trial a circular probe dot appears in a cell of the matrix and participants are asked to indicate as quickly as possible whether the probe falls within the mental image or outside it. Podgorny and Shepard found that the recorded response times on this task did not differ depending on whether the pattern was imagined, remembered, or actually perceived.

A variant of this procedure was developed by Kosslyn and colleagues (Dror & Kosslyn, 1994; Kosslyn, Cave, Provost, & Von Gierke, 1988). On each trial, participants are presented with a blank 4 × 5 matrix and a lowercase letter. Probe items then appear in separate cells of the matrix and participants have to indicate as quickly as possible whether the probes fall within a mental image of the *uppercase* version of letter within the matrix. The pattern of response times using this variant task indicated that imagery for the letters was generated sequentially rather than simultaneously, with complex letters containing more segments requiring longer to imagine than simpler forms. Using this variant Morrison et al. (2011) have shown that socially anxious participants are impaired in their ability to generate imagery for neutral stimuli as compared to non-socially anxious participants (see also Section 4.2.1). This variant has also been used with participants suffering from schizophrenia (Aleman et al., 2005) who show normal performance in comparison to healthy controls, although they were significantly impaired on a further measure of image inspection (see Section 5.3.9).

#### 5.1.2 Mental clocks task

Paivio (1978) reports a task in which participants are verbally presented with pairs of times (i.e., six o'clock and half past five) and asked to indicate which time would produce the smallest angle between the hands if the time was represented by an analogue clock. Response times indicated that participants took longer to make the decision as the angular difference between the two times grew smaller. A variant of the task termed the “O'Clock Test” requires participants to choose the larger rather than smaller angle formed by the hour and minute hands (Grossi, Modafferi, Pelosi, & Trojano, 1989). Again, this task necessitates the generation of mental imagery to provide the correct answer.

#### 5.1.3 Familiar squares description test

A procedure devised by Bisiach and Luzzatti (1978) requires participants to describe a familiar city square from two opposing points of view (for example, the view when standing from the east and then from the west). Performance is scored as the total number of elements from the scene accurately described. Variants of this procedure include describing an imagined map of a country instead of a square (Bartolomeo, D'Erme, & Gainotti, 1994; Rode et al., 2010).

#### 5.1.4 The buildings task

In a task described by Nori and Giusberti (2006) participants are presented with a photograph of a building for 3 s and asked to create a mental image of it. Using this image they are then asked to identify the original picture from three similar distracters. A variant used by Palermo et al. (2010) increases the initial study time from three to ten seconds.

See also the Image Duration Task used by Cocude and Denis (1986, 1988) described below for an additional example of an image generation procedure.

#### 5.1.5 Evaluation of image generation measures

The Image Generation (Section 5.1.1) and Mental Clocks (Section 5.1.2) tasks are computerised measures, while the Familiar Squares (Section 5.1.3) and Buildings (Section 5.1.4) tasks can be implemented without access to a computer. Non-computerised methods may confer the advantage of being easy to administer across a range of different assessment circumstances in clinical settings, and might also be more appropriate for assessing individuals who experience difficulty or are uncomfortable in completing computer-based assessments. However, computerised methods provide higher levels of accuracy in recording behavioural data, including registering image generation times in milliseconds. This greater sensitivity may allow for detection of individual differences that would be more difficult to establish using non-computerised methods alone.

For example, the Image Generation Task (Section 5.1.1) can only be implemented using computerised assessment, and has proved very effective in establishing image generation deficits in clinical groups suffering from social anxiety (Morrison et al., 2011), as well as groups with congenital/prelingual deafness (Emmorey & Kosslyn, 1996). However, this measure only assesses the generation of neutral stimuli, and therefore may fail to detect image generation effects that are specific to emotional material, or highly self-relevant imagery (see discussion in 5.2.1.). For assessing this class of imagery a variant of the Image Duration task described below (see Section 5.2.6) may be more effective, as images are generated in response to concrete nouns rather than neutral stimuli.

### 5.2 Experimental tasks for image maintenance

#### 5.2.1 Image Maintenance task

This task initially devised by Kosslyn, Margolis, Barrett, Goldknopf, and Daly (1990) requires participants to memorise a pattern presented within a matrix (e.g., 4 × 5). In the variant reported by Dror and Kosslyn (1994), once a mental image is formed the pattern disappears and after a retention interval of 2500 ms in which participants are asked to retain the image of the pattern, probe items are presented in separate cells of the blank matrix. As with the image generation task described previously, participants have to indicate as quickly as possible whether the probe falls within the mental image of the pattern or not. Dror and Kosslyn (1994) reported that both error rates and response times on the task increased as the complexity of the pattern to be maintained becomes greater. This demonstrates that the amount of information represented in a mental image places proportionally greater load on the image maintenance process.

#### 5.2.2 Visual Patterns Test

This is a widely-used measure of maintenance in visuo-spatial memory developed by Della Sala, Gray, Baddeley, Allamano, and Wilson (1999), Della Sala, Gray, Baddeley, and Wilson (1997). On each trial participants are presented with a matrix in which half the cells have been filled. After viewing the pattern for three seconds participants are asked to reproduce it accurately using a blank matrix. Three different patterns are presented at each level of complexity, beginning with a simple 2 × 2 matrix and leading up to a 5 × 6 matrix that comprises fifteen filled cells. Performance is scored as being the number of cells in the largest matrix that a participant can accurately recall. Performance on the task has been linked to the maintenance of visual mental imagery (Kemps & Andrade, 2012).

#### 5.2.3 Change detection tasks

Computerised change detection tasks are used to estimate the capacity of visual short-term memory (Luck & Vogel, 1997). Participants judge whether a test array of visual items (e.g. coloured squares) differs (‘change’) or does not differ (‘no change’) from a comparison array presented following a brief retention period. Test and comparison arrays are either identical or differing by a feature of one item (e.g. colour, location). Proportion correct judgments, sensitivity (d′) and estimates of the maximum number of complete items encoded (e.g. Cowan's K) are then calculated.

#### 5.2.4 Visual short-term memory precision

A procedure developed by Bays and Husain (2008) examines the precision of visual short-term memory. In precision paradigms, participants typically view an array of items, e.g. several bars that differ in colour and orientation. Following a short delay, participants reproduce a given feature of one of the items (e.g. orientation) using the method of adjustment. Precision is calculated as the reciprocal of the standard deviation of error in response. Interestingly, precision depends on the total number of items presented (Bays, Catalao, & Husain, 2009; Bays & Husain, 2008). This suggests that visual short-term memory relies upon a limited, dynamic resource that is flexibly distributed among stored items. If more items are encoded in an image, their ‘grain’ or resolution should decline.

#### 5.2.5 Complex span tasks

In the developmental literature, complex span tasks are used to measure active maintenance by the central executive component of working memory (Alloway, Gathercole, & Pickering, 2006). For example, in the odd-one-out task (Russell, Jarrold, & Henry, 1996), participants view three shapes in a single row of a matrix (e.g. 4 x 3), and are asked to identify the odd-one-out. This is repeated until all rows have been probed. At the end of the trial, the participant recalls the location of each odd-one-out shape in order by pointing sequentially to the correct cell in each row on the matrix. While it is clear that such tasks rely upon active executive maintenance, they do not control for the extent to which this maintenance acts upon visual, verbal or some other form of representations (e.g. kinaesthetic).

#### 5.2.6 Image duration tasks

Cocude and Denis (1986, 1988) report studies in which the image duration task requires participants to generate mental images in response to a series of concrete nouns and then indicate at what point their experience of the images ceases. While high-imagery words were found to be related to faster generation times in comparison to low-imagery words there were no reported differences in image maintenance times between the two classes of words. This task has been used to compare latency and duration of imagery in depressed participants compared to healthy controls (Cocude et al., 1997; see Section 5.4.1).

#### 5.2.7 Evaluation of image maintenance measures

All of the described measures are best implemented in the form of computer-based assessment, although the Visual Patterns test (Section 5.2.2) can be presented in a paper-and-pencil based format (see Della Sala et al., 1997; Della Sala et al., 1999). As with image generation (Section 5.1.5) computer-based tasks may provide greater sensitivity by allowing for greater accuracy in measuring the duration of image maintenance. The Image Duration task (Section 5.2.6) combines measures of both image latency and duration, and has been used to study imagery processes in depression (Cocude et al., 1997). Because imagery is generated and maintained in response to concrete nouns, the Image Duration paradigm affords scope to manipulate the emotionality and self-relevance of stimuli by manipulating the nature of the presented cue words.

Only the Image Maintenance (Section 5.2.1) and Image Duration (Section 5.2.6) tasks explicitly require participants to maintain a conscious mental image. The other tasks assess maintenance processes within visual short-term memory, and therefore should be considered indirect measures of image maintenance. A number of recent studies have demonstrated that performance of visual short-term memory tasks involves mental imagery (Borst, Ganis, Thompson, & Kosslyn, 2012; Borst, Niven, & Logie, 2012; Hamame et al., 2012; Kemps & Andrade, 2012). When considering the growing evidence that maintenance processes in mental imagery and visual short-term memory share a common substrate (see Section 3.2) deficits on these tasks can be seen as indicative of potential image maintenance problems.

### 5.3 Experimental tasks for image inspection

#### 5.3.1 Image scanning tasks

Finke and Pinker (1983) reported a procedure in which participants are presented with a pattern of between three and five randomly positioned dots for 4 s. Following a 2 s retention interval, participants are asked to indicate as quickly as possible whether a displayed arrow is pointing to the previous location of one of the dots or not. Recorded latencies revealed a linear relationship between response time and distance between arrow and dot, such that the greater the distance, the longer it took participants to scan their mental image. A variant devised by Dror et al. (1993) presents a square ring of six cells each side within which three cells are randomly darkened on each trial. Following presentation of an arrow participants indicate as quickly as possible whether it points to a previously filled cell or not. A further variant of the Finke and Pinker paradigm has also been published by Borst and Kosslyn (2010).

#### 5.3.2 Map scanning tasks

In the original study reported by Kosslyn, Ball, and Reiser (1978), this task requires that participants memorise a map containing seven locations at different distances from each other. Following this, on each trial participants are asked to focus on one location on the map and are instructed to imagine a small dot moving from that location to another named location. Latencies revealed a linear relationship between response time and spatial distance between locations; the further the distance, the longer the response time. In a variant devised by Mellet et al. (2002), participants are asked to form mental images of a park or village environment that is learnt either from verbal descriptions or a visually-presented map. On each trial they are presented with the names of two landmarks (e.g., church, school) and asked to imagine scanning from the first location to the second.

#### 5.3.3 Letter corner classification task

This was originally described by Brooks (1968), with variants reported by Baddeley, Grant, Wight, and Thompson (1975) and Farah et al. (1988). On each trial an uppercase letter is presented to participants with one corner marked with an asterisk. Participants are asked to imagine the letter and travel clockwise from the marked corner indicating whether they are on the top or bottom of the figure (“yes”) or neither (“no”). The letters F, G, M, N, W, and Z are typically presented.

#### 5.3.4 Mental size comparisons

This task was originally reported by Moyer (1973) who asked participants to decide which of two animals would generally be larger (e.g., a hamster or a mouse), and found that response times were systematically longer if the animals were of a similar size, compared to when the differences in size would be larger (e.g., a dog and an elephant). This was interpreted as reflecting the use of mental imagery to establish the relative size of compared items. A variant was reported by Paivio (1975) that involved size comparisons for both animals and objects (e.g., a table and a bed). Kosslyn, Murphy, Bemesderfer, and Feinstein (1977) have argued that mental imagery is only utilised during mental comparisons when relative size information is not explicitly available in semantic memory; e.g., comparing a tiger and a leopard is more likely to require imagery than comparing an elephant and a fly. A study by Konkle and Olivia has shown that real-world objects are drawn, imagined and preferentially viewed at a consistent visual size (Konkle & Oliva, 2011). There is no standardised list of items for mental size comparisons, but suitable stimuli are described in Paivio (1975), as well as Policardi et al. (1996).

#### 5.3.5 Animal tails test

This task has been described by Farah, Hammond, Mehta, and Ratcliff (1989), Behrmann, Moscovitch, and Winocur (1994), and Policardi et al. (1996). Participants are presented with the names of animals (e.g., kangaroo, pig, elephant) and have to indicate whether they have a long tail proportional to their body size. There is no standardised list of animals used, although animals whose tail is a distinct feature (e.g., beaver, peacock) are avoided. Performance is scored as the number of trials correctly identified.

#### 5.3.6 Straight/curved letter task

In the original task reported by Coltheart, Hull, and Slater (1975) participants are required to imagine the letters of the alphabet sequentially from A to Z in uppercase. External aids such as speaking aloud and writing are not permitted and the task for the participant is to count the number of letters containing a curve. Participants are asked to perform the task as quickly as possible. The outcome measures are time taken to complete the task and the accuracy (correct response versus incorrect response). In a variation reported by Policardi et al. (1996) and later by Bridge, Harrold, Holmes, Stokes, and Kennard (2012), participants are read aloud 20 letters in random order, asked to imagine the presented letter in uppercase, and then to indicate whether the letter contains curves or not. The outcome measure in this variation is the total number of correct responses.

#### 5.3.7 Mental hue comparison task

De Vreese (1991; Expt. 3) reports a task in which participants are verbally provided with pairs of colour-specific objects (e.g., “ripe tomato–ripe apricot; blood–poppy field”) by the experimenter. The task of the participant is to state whether or not they are chromatically similar and the outcome is the number of correct responses.

#### 5.3.8 Top/Bottom larger letter task

Policardi et al. (1996) report a task in which uppercase letters which are larger at either the top or at the bottom (e.g., F, L, V) are read aloud by the experimenter in random order. The participant is required to imagine each letter in upper-case form and then to classify it as being top-larger or bottom-larger. The outcome is the number of correct responses.

#### 5.3.9 Objects form task

This task was first reported by Mehta, Newcombe, and Dehaan (1992). Participants are presented with the names of three objects (e.g., “pumpkin, lettuce, tomato”) and are asked to indicate the odd-one-out based on inspecting the imagined form of the objects (e.g., “lettuce” in the previous example). Aleman et al. (2005) have reported that people with schizophrenia are impaired on this task in comparison to healthy controls. Blind participants have also been shown to make more errors on the objects form task in comparison to sighted controls (Noordzij, Zuidhoek, & Postma, 2007).

#### 5.3.10 Evaluation of image inspection measures

Mental comparison tasks such as relative size (Section 5.3.4), tail length (Section 5.3.5), and colour hue (Section 5.3.7) have been widely used to assess imagery in the literature. They have the advantage of being easy to administer and score, and do not require access to a computer. However, there can be ambiguity in establishing whether task performance is based on inspecting mental imagery or instead abstract semantic knowledge of the items being compared (Holyoak, 1977). Tasks based on mentally inspecting imagery for letters of the alphabet (Sections 5.3.3, 5.3.6, and 5.3.8) may therefore provide more reliable data. The Objects Form task (Section 5.3.9) is also straight-forward to administer and has been linked to evidence of significant impairment in schizophrenia (Aleman et al., 2005) and cases of late-onset blindness (Noordzij et al., 2007).

Map scanning (Section 5.3.2) and image scanning (Section 5.3.1) are computer-based paradigms that may confer greater sensitivity by recording both response time and accuracy scores for participants' imagery-based judgments. Image scanning could be considered preferable to map scanning as it is less prone to accusations that judgments reflect tacit knowledge rather underlying properties of mental imagery (see discussion in Section 3.3). Mental scanning tasks have revealed significant deficits in image inspection related to aging (Brown, Kosslyn, & Dror, 1998) and blindness (Iachini & Ruggiero, 2010). A variant of image scanning based on body width has successfully been applied to evaluate visual disturbances in body image associated with anorexia nervosa (Smeets, Klugkist, van Rooden, Anema, & Postma, 2009).

### 5.4 Experimental tasks for image transformation and manipulation

#### 5.4.1 Mental rotation

##### 5.4.1.1 Classic image rotation tasks

First devised by Shepard and Metzler (1971), these tasks require participants to view pairs of three-dimensional abstract shapes. Participants are then asked to decide whether the pairs are identical or different. Response time has been found to increase with the degree of angular rotation between the two shapes; the further the shapes are rotated from their original position, the longer it takes participants to judge whether the pairs are the same or different. Variants have included rotation of two-dimensional shapes (Cooper, 1975), letters of the alphabet (Cooper & Shepard, 1973), and matrix patterns (Dror & Kosslyn, 1994). A computerised version of the Shepard and Metzler (1971) task was used by Wright, Thompson, Ganis, Newcombe, and Kosslyn (2008). The Cooper and Shepard (1973) task has been used to demonstrate impairment in mental rotation in unipolar depression (Rogers et al., 2002).

##### 5.4.1.2 Manikin test

In this task devised by Ratcliff (1979), participants view a series of drawings of a manikin presented in different rotated positions and are asked to decide as quickly as possible whether a black disk is held in the right or left hand. Thirty-two trials are presented using orientations of forwards-upright, backwards-upright, forwards-inverted, and backwards-inverted. In half the stimuli the black disk is in the right hand and in the other half the left. Performance is quantified as the total number of trials correctly completed.

##### 5.4.1.3 Rotation-arrow task

Devised by Shah and Miyake (1996) this task combines mental rotation with short-term memory for the orientation of a sequence of arrows. On each trial participants are presented with a rotated letter (from F, J, L, P, and R) which they classify as normal or a mirror-image, followed by an arrow pointing in a random orientation. On each trial a set size of between two to five letters can be presented, with performance based on correct recall of the orientation of the sequence of arrows.

#### 5.4.2 Image restructuring and reinterpretation

##### 5.4.2.1 Component detection task

A procedure first reported by Reed and Johnson (1975) in which participants are asked to detect the presence of shapes within patterns that are either visually perceived or imagined. A variant devised by Verstijnen, Hennessy et al. (1998), Verstijnen, van Leeuwen et al. (1998) manipulates whether the shapes required to be detected within the patterns are existing or novel component parts.

##### 5.4.2.2 Interpretation of ambiguous figures

Chambers and Reisberg (1985) report a paradigm in which participants are required to form a mental image of the ambiguous duck/rabbit figure based on a brief visual presentation of five seconds. Participants are then asked to reinterpret the figure based on either mental imagery alone or a sketch drawn from memory. Findings showed that the mental images had been interpreted and were not held ‘ambiguously’. A later variant of this procedure requires participants to mentally rotate a pattern into a position where it can be reinterpreted as a meaningful shape (Mast & Kosslyn, 2002; Reisberg & Chambers, 1991).

##### 5.4.2.3 Image combination and subtraction

Two related imagery reinterpretation procedures have been developed by Brandimonte, Hitch, and Bishop (1992a,b,c). In the *image combination* task participants are sequentially presented with two line drawings which if mentally combined together form a recognisable object (e.g., a skipping rope, a butterfly, a car, etc.). In the *image subtraction* task the procedure is similar, but in this case the second line drawing must be mentally removed from the first in order to form a recognisable object. A full list of the stimuli used is reproduced in Brandimonte et al. (1992c).

#### 5.4.3 Mental synthesis

##### 5.4.3.1 Image combination

Finke et al. (1989) report a procedure in which participants are required to mentally manipulate alpha-numeric symbols in response to a series of verbal instructions. For example, “Imagine a capital ‘D’. Rotate the Fig. 90° to the left. Now place a capital letter ‘J’ at the bottom. Identify the final figure (answer: an umbrella)”. A variant of the procedure is described by Behrmann et al. (1994) in which participants follow verbal instructions in order to transform one letter of the alphabet into another (e.g., “Take the letter ‘V’, turn it upside down. Put a horizontal line through the middle of it” (answer: the letter ‘A’).

##### 5.4.3.2 Creative synthesis

In this procedure devised by Finke and Slayton (1988), participants are verbally presented a series of trials consisting of three alpha-numeric or geometric shapes (e.g., circle, capital ‘D’, number ‘8’). The task requirement is to mentally combine the stimuli into a recognisable object or scene (e.g., a smiling face). The size, position, and orientation of the items can be transformed in any way, although the final design must only include the shapes which were originally specified. Successful trials are verbally labelled and then recorded on a sheet of paper. Variants of the procedure are described by Anderson and Helstrup (1993), Helstrup and Anderson (1996), and Pearson et al. (1999).

##### 5.4.3.3 Temporal image integration task

This image integration task was devised by Brockmole, Wang, and Irwin (2002) from a visual memory task first presented by Di Lollo (1980). On each trial participants are sequentially presented with two dot arrays enclosed within 4 × 4 grids. If both arrays are considered together all cells within the grid would be filled by dots except one. Participants are asked to mentally integrate a visual image of the first array with the perceived second array and then indicate as quickly as possible which cell would remain unfilled. In a variant of the task reported by Lewis, Borst, and Kosslyn (2011) participants are asked to generate the image of the first array either from short-term or long-term memory.

#### 5.4.4 Evaluation of image transformation and manipulation measures

These measures incorporate aspects of image generation, maintenance, and inspection processes. The greater task complexity in comparison to the measures described previously (in Sections 5.1 to 5.3) may mean interpretation of any observed deficits or enhancements in performance are more difficult. However, we propose that evaluation of these processes can be important because image transformation and manipulation underlie clinical techniques such as “imagery restructuring” or “imagery re-scripting”; for example as used in Cognitive Behaviour Therapy (e.g. Holmes, Arntz et al., 2007) or schema focussed therapy (e.g. Giesen-Bloo et al., 2006) and so forth.

Mental rotation tasks (Section 5.4.1) are the most straight-forward to administer, and can be implemented in both computerised and paper-and-pencil variants (with performance in the latter based on the number of items successfully completed within a set interval). Mental rotation processes have been linked to imagined perspective transformations, in which an image is viewed from either an observer (third-person) or field (first-person) perspective (Gardner, Sorhus, Edmonds, & Potts, 2012). Libby, Valenti, Pfent, and Eibach (2011) have shown that imagining failure from an observer rather than field perspective is associated with increased feelings of shame and negativity of accessible knowledge amongst low self-esteem individuals. Mental rotation deficits have been identified in disorders including unipolar depression (Rogers et al., 2002), schizophrenia (Quee, Eling, van der Heijden, & Hildebrandt, 2011), and Williams Syndrome (Stinton, Farran, & Courbois, 2008). Bulimia nervosa patients have also been found to be impaired on mental transformations of their own body, but not on transformations of external objects (Urgesi et al., 2011). Observer perspective is also associated with poor outcomes in PTSD (McIsaac & Eich, 2004) and depression (Kuyken & Moulds, 2009).

Restructuring and synthesis measures (Sections 5.4.2 and 5.4.3) can provide further means to assess an individual's ability to both manipulate and reinterpret mental imagery. Deficits in these areas may impact negatively on the effectiveness of clinical techniques that are based on image manipulation and reinterpretation, and this remains to be further examined. For example, Holmes et al. have argued that imagining positive events from a field perspective may be critical for improving positive emotion (Holmes, Coughtrey, & Connor, 2008). Patients who do display significant deficits in image manipulation and reinterpretation may benefit from access to external support that reduces their requirement to rely on mental visualization (for discussion of cognitive benefits of external support see Barquero & Logie, 1999; Verstijnen, van Leeuwen et al., 1998).

### 5.5 Measures of general imagery use and experience

#### 5.5.1 Betts' Questionnaire Upon Mental Imagery

The original 150-item questionnaire investigates the ability to image across seven sensory modalities (visual, auditory, cutaneous, kinaesthetic, gustatory, olfactory and organic) (Betts, 1909). In the visual modality, for example, participants are asked to think of seeing ‘the sun sinking below the horizon’, and provide a rating for vividness on a 7-point scale, ranging from 1 ‘perfectly clear and vivid’ to 7 ‘no image present at all’. The ‘organic’ modality corresponds to somatic sensations such as hunger and thirst. Although comprehensive, this measure is often considered prohibitively long, and thus, a shortened form has been developed consisting of a subset of 35 items from the original questionnaire (five items from each sensory modality) (Sheehan, 1967). The validity of the shortened form of the questionnaire (also known as the Questionnaire on Mental imagery; QMI) is supported by a high correlation (*r* = .92) with the original scale (Sheehan, 1967).

The QMI has been used by Oertel et al. (2009) to investigate the relationship between the vividness of imagery and predisposition towards hallucinatory experience in schizophrenia (see Section 5.3).

#### 5.5.2 Vividness of mental imagery (VVIQ)

Vividness of mental imagery can be assessed using this battery of 16 questions (Marks, 1973). Participants are asked to generate four mental images in turn (e.g., ‘Visualise the rising sun. Consider carefully the picture that comes before your mind's eye’). For each image, participants respond to four items relating to that image (e.g., ‘The sky clears and surrounds the sun with blueness’) by providing a rating on a scale from 1 ‘perfectly clear and as vivid as normal vision’ to 5 ‘no image at all, you only “know” that you are thinking of an object’. Interestingly, performance on this subjective measure of vividness has been shown to correlate with objective measures of early visual cortex activity as assessed using fMRI as well as performance on a psychophysiological colour-naming task (Cui, Jeter, Yang, Montague, & Eagleman, 2007).

The VVIQ has also been used to explore the relationship between vividness of imagery and schizotypal traits in an analogue non-clinical population (Bell & Halligan, 2010; see Section 4.3).

#### 5.5.3 Spontaneous Use of Imagery Scale (SUIS)

This measure of tendency towards general imagery use was developed by Kosslyn and colleagues (Reisberg, Pearson, & Kosslyn, 2003). It consists of 12 items, such as ‘When I first hear a friend's voice, a visual image of him or her almost always comes to mind’. Participants are asked to read the 12 items and to ‘indicate the degree to which each is appropriate for them’ using a 5-point scale (with 5 indicating ‘completely appropriate’ and 1 indicating ‘never appropriate’). Reisberg et al. (2003) reported good internal consistency for SUIS and a clear relationship between SUIS and VVIQ, with a small but reliable difference for SUIS score between low-imagers and high-imagers, suggesting that the two measures assess a related construct.

In bipolar disorder, significantly higher SUIS scores have been observed compared to healthy volunteers (Holmes et al., 2011) and unipolar depressed participants (Hales et al., 2011), consistent with the clinical hypothesis that people with bipolar disorder have an enhanced propensity to engage in mental imagery; see Section 4.5.

#### 5.5.4 Tellegen Absorption Scale (TAS)

Absorption is defined as ‘openness to absorbing and self-altering experiences’ (Tellegen & Atkinson, 1974). This 34-item scale forms part of the larger Multidimensional Personality Questionnaire (Tellegen, 1982) and assesses predisposition to become highly involved in imaginative or sensory everyday experiences. Items such as ‘If I wish, I can imagine (or daydream) some things so vividly that they hold my attention in the way a good movie or story does’ are rated as ‘true’ or ‘false’ by the participant. Tellegen (1982) reports high levels of internal consistency (*r* = .88) and test–retest reliability (*r* = .91) for this measure in non-clinical samples.

#### 5.5.5 Verbaliser–Visualiser Questionnaire (VVQ)

The VVQ (Richardson, 1977) consists of 15 items taken from Paivio's Ways of Thinking Questionnaire (Paivio, 1971). Eight items tap into visual processing (e.g., ‘My thinking often consists of mental pictures or images’) and seven items tap into verbal processing (e.g., ‘I enjoy doing work that requires the use of words’) to which participants are required to respond using ‘true’ or ‘false’. The outcome measure is a single score, which was later criticised for being one-dimensional in nature. Kirby, Moore, and Schofield (1988) adapted the VVQ such that ten questions assess verbal preference, ten questions assess visual preference and ten questions assess dream vividness (e.g., ‘My dreams are so vivid I feel as though I actually experience the scene’). The revised VVQ involves the calculation of responses to each of the three subscales.

#### 5.5.6 Visualiser–Verbaliser Cognitive Style Questionnaire

This task assesses preference for using imagery as opposed to verbal strategies when problem solving (Kozhevnikov, Kosslyn, & Shephard, 2005). In Part I of the task, participants are required to complete 11 written mathematical problems, taken from the Mathematical Processing Instrument as described in Lean and Clements (1981). An example problem is ‘In an athletics race, Jim is four feet ahead of Tom and Peter is three feet behind Jim. How far is Peter ahead of Tom?’ In Part II, participants are presented with a selection of common strategies for completing each of the problems (e.g., ‘I solved the problem by imagining Jim, Peter, and Tom running in an athletics race’). Participants are asked to indicate as many of the cognitive strategies provided that they utilised in solving each problem, and to describe any further methods they had used.

#### 5.5.7 Predominance of verbal and imagery-based thoughts visual analogue scale (VAS)

Holmes, Mathews et al. (2008) developed three simple rating questions to assess the use of imagery vs. verbal thought (i.e., < during the experimental task > ‘how much did you find yourself thinking in mental images’; ‘how much did you find yourself thinking in words or sentences’ and ‘how much did you find yourself thinking in a manner that neither seemed like mental images nor verbal thoughts’. Responses are made on a 9-point scale, from 1 ‘not at all’ to 9 ‘totally’. As such, this measure allows the collection of convergent self-report data alongside experimental data.

A variant of the task is described by Holmes et al. (2011) in which participants were asked how much their thinking over the past seven days has taken the form of mental images and then asked about verbal thoughts. Responses to each are rated on a scale from 1 (“none of the time) to 9 (“all the time”). Patients with bipolar disorder have shown significantly higher ratings on the imagery processing item compared to healthy volunteers and in contrast, significantly lower ratings on the verbal processing item. This is comparable with similar findings observed with the SUIS (Section 5.5.3).

#### 5.5.8 Mental imagery interview

This is a semi-structured interview developed to explore the subjective experience, occurrence and content of mental imagery in healthy volunteers and clinical populations (Day, Holmes, & Hackmann, 2004) inspired by early studies by Ann Hackmann and colleagues (e.g. Hackmann, Surawy, & Clark, 1998). Since being used in agoraphobia (Day et al., 2004), the core interview has been adapted for use in a variety of clinical groups based on the specific characteristics of imagery of interest. Interviews are audio-taped, transcribed and coded post-interview.

The interview has been used to explore the frequency and nature of images in social phobia (Hackmann et al., 2000) as well as other disorders. Following provision of definition of mental imagery, participants are asked to recall a social situation in which they felt anxious from approximately six months prior to interview and are asked about associated images and the recurrence of such images. Participants then respond to a series of questions relating to the sensory content, visual characteristics, associated emotions, context and meaning of a typical social phobia-related recurrent image from that time. The interview then proceeds to explore the relationship of that image to particular memories through a series of questions. Finally, participants rate how similar that image is to a particular memory on a 0–100 scale in relation to sensory and then interpersonal content. All participants reported recurrent negative images, most of which were related to an adverse social event occurring near to the time of onset of the disorder suggesting a relationship with onset and maintenance of the disorder (Hackmann et al., 2000).

An adaptation of the interview has been used to assess the relationship between mental imagery and psychotic symptoms in schizophrenia (Morrison et al., 2002). Patients are asked to evoke a previously experienced image and the content, occurrence and meaning of the image, including associated emotions, memories and beliefs, are examined using the interview. Findings from Morrison and colleagues confirmed that the majority of patients (approximately 75%) experienced mental images in relation to their delusions and hallucinations. Further, most of these were recurrent and associated with affect, beliefs and memories, suggesting a new avenue for mental imagery in understanding psychosis.

The interview has also been used to explore the frequency and nature of suicidal imagery in depression (Holmes, Crane et al., 2007). The interview begins by defining mental imagery and then a checklist is used to assess the content of cognitions when participants were at their most despairing. Participants then identify the most significant image they have experienced when at their most despairing/suicidal and describe this in detail, and are asked about associated affect and meaning. Distress and comfort are rated on scales from 1 “not at all” to 9 “extremely” and participants also rate how real the images felt and their preoccupations with the images, again using nine-point scales. Results showed that all participants experienced intrusive, recurrent suicide-related images when at their most despairing, which the authors termed “flash-forwards” to suicide (replicated by Crane et al., 2012; Hales et al., 2011). Hales et al. (2011) have further applied the interview to investigate imagery relating to suicidal cognitions in both bipolar and unipolar patients (see Section 4.5.1).

#### 5.5.9 Controllability of Visual Imagery Questionnaire

Developed and reported by Richardson (1994), this questionnaire assesses the ability to visualise and manipulate a given scenario in response to set cues. Participants are asked a series of questions (e.g., “Can you see a car standing on the road in front of a house”, then “Can you see < the car > climb up a very steep hill”). For each of the twelve items, participants respond with “yes”, “unsure” or “no”.

#### 5.5.10 Observer vs. field visual analogue scale (VAS)

This scale has been used to assess the perspective from which an image relating to a previously experienced social situation is experienced (Hackmann et al., 1998; Wells, Clark, & Ahmad, 1998). Perspective of the image is rated by the participant on a 7-point scale, ranging from − 3 (“field perspective”) to + 3 (“observer perspective”). Findings in these two studies confirmed that in contrast to healthy volunteers, individuals with social phobia typically adopt an observer perspective in images of social situations, viewing themselves as if from an external point of view. A variant of the scale is reported by Stopa and Bryant (2004) who investigated the perspective of memory and relation to self-concept. In this study, high and low socially-anxious individuals reported memories of social occasions and designated the perspective of these memories as “observer”, “field”, or “neither”. In comparison to previous findings, in this study both high and low anxious participants predominately adopted a field perspective, that is, “as if seeing the situation through their own eyes”.

#### 5.5.11 Evaluation of measures of general imagery use and experience

An important consideration when assessing mental imagery ability is whether to adopt objective or subjective measures. We recommend where possible that both types of method are employed, as they assess unique and separable dimensions of imagery ability. Zeman et al. (2010) have reported a case of so-called “blind imagination” in which a patient who reported abrupt cessation of subjective mental imagery was still able to perform normally on many standard imagery measures. This suggests that performance on visual imagery tasks can be dissociated from the phenomenological experience of visual imagery itself. However, as discussed in Section 3.5, there are several fMRI studies reported in the literature which have established a correspondence between the subjective experience of imagery in different modalities (i.e., imaging what something looks like, or sounds like) and activation in brain areas that are associated with perceptual processing in the same modality (Belardinelli et al., 2009; Huijbers et al., 2011). The technique of ‘self-imaging’ (i.e., imagining an event from a realistic and personal perspective) has also been shown to significantly enhance recognition memory in individuals who suffer from neurological memory impairments (Grilli & Glisky, 2010).

There is an abundance of measures to assess the subjective experience of imagery and these have been used in a variety of disorders including schizophrenia, depression and bipolar disorder, but the selection of measures has not been applied consistently, either between or within specific disorders. This has brought some interesting issues to light. For example, two measures of vividness of imagery have been explored in schizophrenia (Sections 5.5.1 and 5.5.2), but conflicting findings led Bell and Halligan (2010) to suggest that if vividness of imagery is indeed trait marker for schizotypy, it is only apparent when imagery is assessed across several domains. Further, some subjective accounts of imagery experience may not fall neatly into imagery or diagnostic categories (for example the experience of synaesthesia; Banissy, Walsh, & Ward, 2009), but may nonetheless be a worthwhile target for further research in clinical populations. We believe that subjective experience presents an important aspect of mental imagery assessment, and that careful consideration of the aspect of interest in specific clinical groups will best inform test selection in this area (see Table 2).

The Predominance of Verbal and Imagery-based Thoughts VAS (Section 5.5.7) has been used with bipolar patients to demonstrate a significant predisposition towards imagery-based processing styles (Holmes et al., 2011). The scale is short and simple to administer, and can provide an effective method for initial investigation of imagery-use in a clinical population. However, to date it has not been validated to the same extent as other more established measures. We therefore recommend that it be used in conjunction with either the VVQ (Section 5.5.5) or the Visualiser–Verbaliser Cognitive Style Questionnaire (Section 5.5.6) which can provide more detailed complimentary information on imagery and verbal processing styles.

Versions of the clinically orientated ‘Mental Imagery Interview’ (Section 5.5.8) have been used in several disorders, including social phobia (Hackmann et al., 2000), agoraphobia (Day et al., 2004), depression (Holmes, Crane et al., 2007), bipolar disorder (Hales et al., 2011), chronic pain (Berna et al., 2011) and mental contamination (Coughtrey, Shafran, Lee, & Rachman, 2012). We suggest that when tailored to the specific disorder, the Mental Imagery Interview provides an appropriate methodology to investigate the occurrence, content and experience of mental imagery in clinical populations, and generates information that can be useful as a therapeutic target. Related approaches have been used in obsessive-compulsive disorder (de Silva, 1986; de Silva & Marks, 1999) and health anxiety (Muse, McManus, Hackmann, & Williams, 2010; Wells & Hackmann, 1993). We propose that exploring the role of imagery in these disorders, and others which have been relatively neglected in this regard, holds promise for future research and possible clinical innovation.

### 5.6 Measures to assess imagery re-experiencing phenomena and intrusions/flashbacks

#### 5.6.1 Script-driven imagery procedures

These procedures are a clinical technique aiming to provoke symptomatic PTSD responses. Participants are typically presented with verbal scripts and asked to imagine the content. This procedure seems to have developed from earlier work using generic scripts targeting common fears including snake phobia and performance anxiety (e.g., Lang, Levin, Miller, & Kozak, 1983). More recently, scripts of previously obtained individual narratives relating to an experienced traumatic event have been used to investigate physiological responses and the effects of pharmacological interventions administered post-trauma (Pitman et al., 2002). Script-driven imagery techniques have also been adapted for use in fMRI studies of PTSD (Lanius et al., 2001; Lanius et al., 2002; Rauch et al., 1996; Shin et al., 1997) allowing the investigation of the neural correlates of trauma response and re-experiencing phenomena.

#### 5.6.2 Impact of Event Scale

The IES (Horowitz, Wilner, & Alvarez, 1979) is a self-report measure anchored to a previously experienced traumatic event aiming to assess avoidance and intrusive symptomatology. The Impact of Events Scale—Revised (IES-R; Weiss & Marmer, 1997) was adapted to include a hyperarousal subscale in order to more closely reflect the PTSD diagnostic criteria of DSM-IV (American Psychiatric Association, 2000). Exploration of the properties of the scale in male Vietnam veterans confirmed a high internal consistency of the overall scale and high correlation between the IES-R and the PTSD Checklist (Weathers, Litz, Herman, Huska, & Keane, 1993) but did not support the three-factor structure of the measure (Creamer, Bell, & Failla, 2003). However, more recent research in a sample of motor vehicle accident survivors confirmed the three-factor structure (i.e., intrusion, avoidance, and hyperarousal subscales) as well as adequate internal consistency for each of the subscales (Beck et al., 2008). The intrusion subscale is that most closely linked to re-experiencing symptoms and thus intrusive imagery.

#### 5.6.3 Intrusion triggering tasks

In line with the observation that real-life perceptual triggers may result in flashbacks in PTSD (Ehlers & Clark, 2000; Ehlers et al., 2004), this laboratory-based methodology is designed to trigger intrusive images in healthy volunteers by presenting perceptual reminders of a previously experienced analogue stressful event, such as a stressful film (for reviews of the Stressful Film Paradigm see Holmes & Bourne, 2008; Holmes, Brewin, & Hennessy, 2004). Participants are presented with a series of neutral static images taken from the stressful film and are then asked to record the number of intrusions experienced under varying experimental conditions within a set timeframe (e.g., Holmes, James et al. 2009; Holmes et al., 2010; Lang, Moulds, & Holmes, 2009).

#### 5.6.4 Intrusion diaries

Tabular paper diaries have been used to record the frequency and content of intrusive images as they occur in daily life (Davies & Clark, 1998; Holmes et al., 2004). Participants are asked to carry the diary with them over a set period of time (e.g., over one week) and record any involuntary intrusive images experienced as well as provide information on the content. Intrusion diaries have been used in healthy volunteers to record involuntary intrusions of a stressful film (e.g., Bourne, Frasquilho, Roth, & Holmes, 2010; Butler, Wells, & Dewick, 1995; Davies & Clark, 1998; Hagenaars, Brewin, van Minnen, Holmes, & Hoogduin, 2010; Holmes, James et al., 2009; Holmes et al., 2004; Krans, Naring, & Becker, 2009; Pearson, 2012; Pearson, Ross, & Webster, 2012; Stuart, Holmes, & Brewin, 2006). A variant of the intrusion diary has been used clinically by therapists to record and detail traumatic “hotspots” (detailed moments of peak emotional distress during a traumatic event) in patients suffering PTSD (Holmes et al., 2005). This work has shown that the majority of intrusions experienced in PTSD are also a hotspot and that these are accompanied by a range of negative emotions (Grey & Holmes, 2008; Holmes et al., 2005).

#### 5.6.5 Intrusions interview

A structured interview investigating intrusive imagery in depression has been developed by Patel et al. (2007) similar to the mental imagery interviews described earlier (Section 5.5.8). Participants are asked to report the total number of autobiographical depressive memories, consisting of a visual image which had repeatedly come to mind over the previous week. The two most frequent and distressing memories are explored further in the interview. These memories are then rated by the participant for emotionality, vividness, sense of “now-ness” and re-experiencing, interference in daily activities, uncontrollability and distress using 0–100 rating scales. The same questions are then repeated for intrusive images, defined as a sensory representation of part of a memory, without surrounding or context, or of an imagined event. Findings indicated that approximately half the depressed patients experienced some form of negative intrusive imagery, and that this interfered significantly with their everyday lives. See also Kuyken and Brewin (1994), Reynolds and Brewin (1999) and Williams and Moulds (2007).

#### 5.6.6 Evaluation of measures to assess imagery re-experiencing phenomena and intrusions/flashbacks

The Impact of Event Scale (Section 5.6.2) and Intrusions Interview (Section 5.6.5) both provide structured methods for evaluating a patient's re-experiencing symptoms and frequency of trauma-related intrusions. However these methods are dependent on a patient's ability to accurately recall their previous image-based intrusions, and therefore may be prone to inaccuracy, bias, or non-disclosure of relevant information. Intrusion diaries (Section 5.6.4) may be more effective as they are typically completed for a longer time period. Again, however, the quality of data collected from diaries is closely related to the degree of compliance by participants with the experimenter/clinician instructions.

Script-driven imagery procedures (Section 5.6.1) and intrusion triggering tasks (Section 5.6.3) confer the advantage of assessing intrusions “as they happen”, but could be regarded as being less naturalistic than intrusion diaries or interviews that focus on re-experiencing phenomena occurring outside of clinical or experimental sessions. Because script-driven imagery procedures are based on verbal scripts and narratives they may reflect linguistic processes that are not directly associated with re-experiencing phenomena and intrusions (Brewin, 2007). In contrast, intrusion triggering tasks based on presenting perceptual reminders of traumatic experiences may more closely model the role played by perceptual environmental cues in the onset of intrusions and flashbacks (see Brewin et al., 1996; Conway & Pleydell-Pearce, 2000; Ehlers & Clark, 2000).

### 5.7 Measures to assess prospective imagery

#### 5.7.1 Impact of Future Events Scale (IFES)

This is an adaptation of the IES-R (Section 5.6.2) created by Deeprose and Holmes (2010), in which each item of the IES-R was adapted to assess intrusive “pre-experiencing” and imagery of specific, future events. When the IFES was explored in a non-clinical sample in relation to risk for bipolarity, IFES Total Score was positively associated with risk for bipolarity (Deeprose, Malik, & Holmes, 2011). In patients with bipolar disorder, significantly higher IFES Total Scores were found compared to healthy volunteers (Holmes et al., 2011). Furthermore, when these bipolar patients were categorised into two groups according to mood stability (stable vs. mood unstable), higher IFES Total Scores were observed in the unstable group (Holmes et al., 2011). Finally, higher IFES Total Scores have been observed for bipolar patients when compared to unipolar depressed controls (Hales et al., 2011). This overall pattern of data suggests that bipolar disorder is indeed associated with high levels of prospective intrusive imagery for personal events (Holmes, Geddes et al., 2008).

#### 5.7.2 Autobiographical memory and future cueing task

D'Argembeau et al. (2008) used an adaption of this task originally reported by Williams et al. (1996) in the context of depression (see Section 4.4). In the memory task, participants are asked to recall events; in the future task participants are asked to imagine events “that might reasonably happen to them” in the future. Both the memory and future task adopt a cueing procedure in which participants are presented with short sentences (e.g., “a situation in which you feel guilty about something”; “a situation in which someone smiles at you”). D'Argembeau and colleagues used positive and negative cues (although the original task reported by Williams and colleagues also included neutral cues). Participants have 60 s to respond with a specific event to each cue. Responses are audiotaped, transcribed then scored. Findings indicated that the participants with schizophrenia recalled fewer past events and future events than healthy controls and that these impairments were associated with the positive symptoms of the disorder. Interestingly, the patients with schizophrenia were even more impaired in generating future events than past events. However, it should be noted that the task does not necessarily require that participants use imagery in order to respond to the cues and thus it is possible that participants also used verbal strategies rather than imagery in the generation of past and future events.

Williams et al. (1996) previously found that suicidal depressed participants demonstrated impairment in generating images of the future for all sets of cues (positive, negative and neutral) compared to matched controls. This degree of impairment was found to correlate with the extent to which participants had difficulty in retrieving specific autobiographical memories.

#### 5.7.3 Prospective imagery task

This task requires participants to form mental images of future events (Holmes, Lang et al., 2008; Stöber, 2000). Participants are provided with set cues of positive and negative scenarios (e.g., “You will have a serious disagreement with your friend”, “You will do well on your course”). In a study by Stöber (2000), non-clinical participants rated each image for detailedness, speed and vividness using VAS. Depression scores were associated with decreased vividness of images for positive future events. These findings were replicated by Holmes, Lang et al. (2008) in which vividness ratings were provided on a scale ranging from 1 (no image at all) to 5 (very vivid). Further, this study compared performance in participants grouped by depression scores and showed that high dysphoric participants reported more vivid negative prospective imagery and less vivid positive prospective imagery than the low dysphorics. A study by Morina et al. (2011) confirmed poorer ability in clinically depressed patients to vividly imagine positive future events using this task compared to healthy age and gender matched controls.

Holmes et al. (2011) used a variant of the prospective imagery task in bipolar disorder that utilised a six-point rating scale for the vividness of each image from 1 (no image at all) to 6 (more vivid than reality) (sixth point added based on pilot work specific to this disorder). This study showed that compared to healthy controls, the bipolar group had higher vividness of negative scenarios, but no difference was observed between groups for positive scenarios (Holmes et al., 2011).

#### 5.7.4 Evaluation of prospective imagery measures

The clinical literature points to the emerging importance of prospective imagery across disorders. Prospective imagery has been considered in relation to schizophrenia, depression and bipolar disorder, with intriguing findings. Imagery for the future is of particular clinical interest, as findings from experimental psychology tells us that imagining oneself completing a behavior may increase the likelihood of that behavior being accomplished (Libby, Shaeffer, Eibach, & Slemmer, 2007). Thus the absence of positive imagery in depression (Holmes, Lang et al., 2008; Morina et al., 2011) and the findings that depression and bipolar disorder are associated with “flash-forwards” to suicide (Hales et al., 2011; Holmes, Crane et al., 2007) are especially concerning (Holmes & Mathews, 2010) but may present viable targets for innovative intervention.

Our review points to several measures for assessing prospective imagery. Voluntary and involuntary cognitive processes can show independence (Conway & Pleydell-Pearce, 2000), though see also Rubin, Boals, and Bernsten (2008). The clinical literature highlights the role of involuntary cognitions not only in PTSD but depression and other disorders. It is thus important to distinguish between measures which reflect controlled, deliberate processes and those which assess involuntary, intrusive cognitions. The Autobiographical Memory and Future Cueing Task (Section 5.7.2) and Prospective Imagery Task (Section 5.7.3) are laboratory tasks which use standardised set cues and require deliberate generation of responses (although note the imagery requirements between the two tasks differ somewhat). In contrast, the Impact of Future Events Scale (Section 5.7.1) attempts to assess real-world personally relevant intrusive imagery. In the future, diary methods may also be better developed. We suggest that researchers should consider both deliberate and involuntary measures in order to fully evaluate prospective cognition in clinical disorders.

### 5.8 Measures used to assess bias in imagery

#### 5.8.1 Cognitive bias modification task

A computerised cognitive bias modification paradigm (CBM; based on Blackwell & Holmes, 2010; Holmes, Lang, & Shah, 2009; Holmes et al., 2006) to explore the effects of imagery-based training in schizophrenia is reported by Steel et al. (2010). This CBM paradigm has been optimised to maximise the use of mental imagery. The task begins with a brief imagery training session (Holmes & Mathews, 2005; Holmes et al., 2006) in which participants are asked to imagine cutting a lemon in order to clarify what is meant by “using mental imagery” and complete practice examples of CBM training. During the CBM training, participants are presented with auditory paragraphs containing a scenario which is initially ambiguous but is ultimately resolved in a positive way. For example: “You are walking down your street and see a gang of children laughing. As you get nearer you see what they are laughing at, *and smile to yourself*” (resolution in italics). Importantly, the initial part of each scenario is ambiguous and could also be resolved with a negative outcome (e.g. *they are laughing at you*). Participants are required to generate mental images of each scenario as though each one is happening to themselves. Thus the aim is to train participants to use mental imagery to generate positive resolutions when confronted with ambiguous information. Change in interpretation bias is assessed by emotional valence ratings of ambiguous test descriptions provided by participants both before and after the CBM training. Although Steel and colleagues did not demonstrate a significant change in interpretation bias following CBM in the overall sample, a significant positive relationship was observed between the self-rated use of imagery in everyday life provided by participants and change in interpretation bias. Thus participants who had a higher tendency to use mental imagery in general were more likely to develop a more positive bias through the CBM training, highlighting the possible importance of trait mental imagery ability in this regard.

Blackwell and Holmes (2010) explored the implementation of repeated CBM training in participants currently experiencing a major depressive episode using a single-case series design. Seven participants completed daily sessions of computerised CBM over a period of one week at home. Findings showed that four of the seven participants demonstrated improvements in mood, bias and/or mental health after one week. Findings were replicated in a larger sample (Lang, Blackwell, Harmer, Davison, & Holmes, 2012). This suggests that the laboratory CBM technique to generate positive mental imagery might hold potential for clinical translation to modify biases and improve mood in depression.

#### 5.8.2 Ambiguous sentences test

Reported by Berna, Lang, Goodwin, and Holmes (2011), this task presents participants with 24 ambiguous scenarios. Participants are asked to form a mental image of the scenario happening to them personally e.g. “You wake up, get out of bed, stretch, and really notice how you feel today.” They then rate the image in terms of how pleasant it is, from 1 “extremely unpleasant” to 9 “extremely pleasant”, and how vivid their image is, from 1 “not at all vivid” to 7 “extremely vivid. High dysphoric participants rated scenarios as less pleasant than a low dysphoric comparison group, suggesting that this is a potential measure of negative interpretation bias in depression.

#### 5.8.3 Homograph interpretation task

Reported by Hertel, Mathews, Peterson, and Kintner (2003), this task was subsequently adapted and used in depression (Holmes, Lang et al., 2008). In this version, participants are asked to form a mental image which includes themselves, based on homographs (e.g., “break” which could be interpreted to mean either broken or rest). After providing a brief description, participants then rate image vividness on a scale from 1 (not at all vivid) to 7 (extremely vivid) and image pleasantness from 1 (extremely unpleasant/negative) to 9 (extremely pleasant/positive). Descriptions of the images are independently rated as positive, negative or ambiguous, based on the context provided by the participant. Vividness and pleasantness scores are calculated separately for the positive and negative responses. The main finding was that high dysphoric participants provided *lower* pleasantness ratings of images generated in response to homographs they interpreted as positive compared to low dysphorics (Holmes, Lang et al., 2008).

On a shortened version of the task patients with bipolar disorder reported significantly fewer positive interpretations of the homographs and a trend towards significantly more negative interpretations compared to healthy volunteers. However, when the patients with bipolar disorder did generate positive interpretations on this task, these images were rated as more vivid than those of the healthy volunteers (Holmes et al., 2011).

#### 5.8.4 Evaluation of measures of bias in imagery

There are a growing number of studies reported in the literature that have demonstrated clinical importance for the interaction between imagery and cognitive bias (e.g. Hirsch et al., 2006). Both the Homograph Interpretation Task (Section 5.8.3), and the Ambiguous Sentences Test (Section 5.8.2) have found performance differences with participants suffering from depression (Berna, Lang, Goodwin, & Holmes, 2011) and bipolar disorder (Holmes et al., 2011). The picture-word cue task, as used by Holmes, Mathews, et al. (2008) similarly asks people to form a mental image combination of a ambiguous picture and an emotionally valenced word and to rate the resultant emotionally valenced mental image. Further development of tasks which may tap into the combination of imagery and an emotional/cognitive bias may be useful in pinpointing psychopathologies.

The application of computerised CBM training (Section 5.8.1) has demonstrated significant improvements from pre-treatment to post-treatment in small scale studies of participants suffering from depression (Blackwell & Holmes, 2010; Lang et al., 2012). However, the exact mechanism of change associated with CBM remains unclear. It could increase the experienced frequency of positive imagery, change the interpretation bias associated with imagery, or a combination of the two. Lang et al. (2012) have suggested that participants may require a certain baseline of mental imagery ability to gain from application of a CBM paradigm. We suggest future research that examines CBM in conjunction with measures of image generation (Section 5.1), inspection (Section 5.3) and transformation (Section 5.4) will be valuable in helping pinpoint the causal mechanisms associated with beneficial effects of imagery-based CBM training.

## 6 Closing remarks: a guiding framework

In this review of mental imagery research in clinical psychology we have considered findings from PTSD, social phobia, schizophrenia, depression and bipolar disorder. We have noted that studies across disorders have rarely used the same measures and the selection of measures has typically been driven by the phenomenology of the disorder in question. Unfortunately, this approach has made it difficult to compare the role of imagery across disorders or to select measures for new research in a previously unstudied disorder. This highlights the need for a guiding framework of the domains most relevant to mental imagery research in clinical psychology.

A wide range of measures exist to assess the four stages of mental imagery (Kosslyn, 1980, 1994; Kosslyn et al., 2006) as reviewed in Section 2. Our review of the clinical literature demonstrates that these are very rarely utilised in clinical research but are a potential source of valuable data. For example, it has been shown that depressed patients are not impaired in image duration (Cocude et al., 1997) but that general sensory/motor processing may be the locus of impairment (Zarrinpar et al., 2006). The robustness of mental imagery *per se* provides a suitable basis for pursuing imagery as a treatment target in this population, e.g., in Cognitive Behavioural Therapy (Holmes, Lang, & Deeprose, 2009; Patel et al., 2007; Williams & Moulds, 2007).

These findings support our argument that there may be aspects of imagery associated with clinical disorders which may be informative to consider from a cognitive point of view. For example, we have seen that bipolar disorder is associated with high imagery use in line with predictions (Holmes, Geddes et al., 2008). However, recent findings from Pan et al. (2011) suggest that remitted bipolar patients show deficits in visuospatial working memory under high working memory load conditions. How these cognitive deficits relate to the phenomenology of imagery in bipolar disorder remains unexplored. This illustrates the need to further explore bipolar and other disorders in relation to the experimental measures of imagery performance. We suggest that in order to further understand imagery in clinical disorders, we should explore the underlying cognitive processes based on a theory-driven and experimentally informed approach.

In summary, we have proposed several key domains for mental imagery assessment in clinical research (see Table 4). This guiding framework incorporates the findings from our review in relation to the cognitive, subjective, and clinical measures of mental imagery, and presents those domains which we believe will be of most interest to clinicians. The guiding framework highlights the broad domains shown to be important in research to date. In selecting tests, consideration will of course need to be given to the specific disorder in question, as well as the pragmatic issues related to using experimental tasks in any given clinical population. For example, experimental tasks may need to be adapted or shortened so as not to fatigue participants (e.g., Zarrinpar et al., 2006). Specific groups of patients may need further adaptations to the measures proposed, such as in the case of neuropsychological impairment (e.g., Bridge et al., 2012). It is also important when assessing mental imagery with clinical populations that performance on more than one imagery measure is examined at a time, and that performance is compared with levels achieved by the same group on appropriate control measure(s). This will help determine whether any observed deficit in task performance is driven by specific imagery impairment or more generalised cognitive dysfunction (see Section 4 for further discussion on this point).

We believe that thorough assessment of mental imagery as proposed here by our guiding framework will help move forward our understanding of the underlying mental imagery processes across a range of psychological disorders, and help drive forward advances in both theory and treatment. We look forward to continued growth and revision of such tasks, and the clinical questions concerning where and why they might best be deployed, as this young field continues to grow.

## Figures and Tables

**Table 1:** Summary of experimental tasks to assess the four cognitive stages of mental imagery (Kosslyn, 1980, 1994; Kosslyn et al., 2006).

Domain	Task	Author	Format
*Generation*	Image Generation Task	Original: Podgorny and Shepard (1978) Variant: Kosslyn et al. (1988) Variant: Dror and Kosslyn (1994)	Computerised
	Mental Clocks Task	Original: Paivio (1978) Variant: Grossi et al. (1989)	Verbal/pen and paper
	Familiar Squares Description Test	Original: Bisiach and Luzzatti (1978) Variant: Bartolomeo et al. (1994) Variant: Rode et al. (2010).	Verbal/pen and paper
	Buildings Task	Original: Nori and Giusberti (2006) Variant: Palermo et al. (2010)	Verbal/pen and paper
*Maintenance*	Image Maintenance Task	Original:Kosslyn et al. (1990); Dror and Kosslyn (1994)	Computerised
	Visual Patterns Test	Original: Della Sala et al. (1997, 1999)	Computerised/pen and paper
	Change Detection Task	Original: Luck and Vogel (1997)	Computerised
	Visual Short-Term Memory Precision	Original: Bays and Husain (2008) Variant: Bays et al. (2009)	Computerised
	Complex Span Tasks	Original: Russell et al. (1996) Variant: Alloway et al. (2006)	Computerised
	Image Duration Tasks	Original: Cocude and Denis (1986, 1988)	Computerised/pen and paper
*Inspection*	Image Scanning Task	Original: Finke and Pinker (1983) Variant: Dror et al. (1993) Variant: Borst and Kosslyn (2010)	Computerised
	Map Scanning Tasks	Original: Kosslyn et al. (1978) Variant: Mellet et al. (2002)	Computerised/ pen and paper
	Letter Corner Classification Task	Original: Brooks (1968) Variant: Baddeley et al. (1975) Variant: Farah et al. (1988)	Pen and paper
	Size Comparison of Paired Animals Task	Original: Moyer (1973)	Computerised/pen and paper
	Animals' Tail Test:	Original: Farah et al. (1989); Behrmann et al. (1994)	Pen and paper
	Straight/Curved Letter Task	Original: Coltheart et al. (1975) Variant: Policardi et al. (1996); Holmes et al. (2011)	Pen and paper
	Mental Hue Comparison Task	Original: De Vreese (1991)	Pen and paper
	Top/Bottom Larger Letter Task	Original: Policardi et al. (1996)	Pen and paper
	Objects Form Task	Original: Mehta et al. (1992) Variant: Aleman et al. (2005); Noordzij et al. (2007)	Pen and paper
*Transformation and Manipulation: Mental Rotation*	Image Rotation Task	Original: Shepard and Metzler (1971) Variant: Cooper (1975) Variant: Cooper and Shepard (1973) Variant: Dror and Kosslyn (1994) Variant: Wright et al. (2008)	Pen and paper/computerised
	Manikin Task	Original: Ratcliff (1979)	Pen and paper
	Rotation Arrow Span Task	Original: Shah and Miyake (1996)	Computerised
*Transformation and Manipulation: Rrestructuring and Reinterpretation*	Component Detection Task	Original: Reed and Johnson (1975) Variant: Verstijnen, Hennessy et al. (1998); Verstijnen, van Leeuwen et al. (1998)	Computerised
	Interpretation of Ambiguous Figures	Original: Chambers and Reisberg (1985) Variant: Mast and Kosslyn (2002); Reisberg and Chambers (1991)	Pen and paper
	Image Combination and Subtraction	Original: Brandimonte et al. (1992a,b,c)	Pen and paper
*Transformation and Manipulation: Mental Synthesis*	Image combination	Original: Finke et al. (1989) Variant: Behrmann et al. (1994)	Pen and paper
	Creative synthesis	Original: Finke and Slayton (1988) Variant: Anderson and Helstrup (1993) Variant: Helstrup and Anderson (1996) Variant: Pearson et al. (1999)	Pen and paper
	Temporal Integration Task	Original: Brockmole et al. (2002) Variant: Lewis et al. (2011)	Computerised

**Table 2:** Summary of tasks to assess general imagery use and experience.

Domain	Task	Author
*Experience, content, and occurrence of imagery*	Betts' Questionnaire upon Mental Imagery Short-form (QMI)	Betts (1909) Sheehan (1967)
	Vividness of Visual Imagery Questionnaire (VVIQ)	Marks (1973)
	Spontaneous Use of Imagery Scale (SUIS)	Reisberg et al. (2003)
	Tellegen Absorption Scale (TAS)	Tellegen (1982)
	Mental Imagery Interview	Original: Hackmann et al. (2000) Variant: Morrison et al. (2002) Variant: Day et al. (2004) Variant: Holmes, Crane et al. (2007) Variant: Hales et al. (2011) Variant: Crane et al. (2012)
*Controllability of imagery*	Controllability of Visual Imagery Questionnaire	Richardson (1994)
*Perspective*	Observer vs. Field Visual Analogue Scale (VAS)	Hackmann et al. (1998) Wells et al. (1998) Stopa and Bryant (2004)
*Imagery versus verbal processing style*	Verbaliser–Visualiser Questionnaire (VVQ) Revised VVQ	Richardson (1977) Kirby et al. (1988)
	Visualiser–Verbaliser Cognitive Style Questionnaire	Kozhevnikov et al. (2005)
	Predominance of Verbal and Imagery-based Thoughts Visual Analogue Scale (VAS)	Holmes, Mathews, Mackintosh, and Dalgleish (2008)

**Table 3:** Summary of tasks used to assess clinical aspects of imagery.

Domain	Task	Author	Format
*Re-experiencing*	Script driven imagery procedures	Original: Lang et al. (1983) Variant: Pitman et al. (2002) Variant: Lanius et al. (2001, 2002) Variant: Rauch et al. (1996) Variant: Shin et al. (1997)	Verbal
*Intrusions/flashbacks*	Impact of Event Scale	Original: Horowitz et al. (1979)	Pen and paper
	Impact of Event scale—Revised	Original: Weiss and Marmer (1997)	Pen and paper
	Intrusion Triggering Task	Original: Holmes, James, Coode-Bate, and Deeprose (2009) Variant: Lang et al. (2009)	Computerised
	Intrusion Diaries	Original: Davies and Clark (1998) Variant: Holmes et al. (2004) Original: Holmes et al. (2005)	Pen and paper Verbal
	Intrusions Interview	Orignal: Patel et al. (2007)	Verbal
*Prospective Imagery*	Impact of Future Events Scale	Original: Deeprose and Holmes (2010)	Pen and paper
	Autobiographical Memory and Future Cueing Task	Original: Williams et al. (1996) Variant: D'Argembeau et al. (2008)	Verbal
	Prospective Imagery Task	Original: Stöber (2000) Variant: Holmes, Lang, Moulds, and Steele (2008)	Pen and paper
*Bias in Imagery*	Cognitive Bias Modification Task	Original: Holmes et al. (2006) Variant: Holmes, Lang, and Shah (2009) Variant: Blackwell and Holmes (2010) Variant: Steel et al. (2010) Variant: T.J. Lang et al. (2012)	Computerised
	Ambiguous Sentences Test	Original: Berna, Vincent, et al. (2011)	Computerised
	Homograph Interpretation Task	Original: Hertel et al. (2003) Variant: Holmes, Lang et al. (2008) Variant: Holmes et al. (2011)	Pen and paper/Computerised

**Table 4:** Summary of domains of mental imagery for assessment in clinical psychology.

Aspect of imagery	Domain for assessment
Cognitive aspects of imagery	Generation Maintenance Inspection Transformation and manipulation
General imagery use and experience	Experience, content, and occurrence Controllability Perspective Imagery vs. verbal processing style
Clinical aspects of imagery	Re-experiencing Intrusions/flashbacks Prospective Imagery Bias in imagery
